# An Efficacy- and In Vivo Exposure-Oriented Integrated Study to Investigate the Effective Components of *Qishen* Granule

**DOI:** 10.3390/ph18101584

**Published:** 2025-10-20

**Authors:** Yueting Li, Tengteng Wang, Chao Cheng, Yingying Huo, Ying Tan, Yifan Xu, Jiale Gao, Jie Liu, Hongbin Xiao

**Affiliations:** 1 School of Chinese Materia Medica, Beijing University of Chinese Medicine, Beijing 100029, China; yuetingli1111@163.com (Y.L.); wtt202304@163.com (T.W.); chengchao0708@163.com (C.C.); hying1229@163.com (Y.H.); 15773239598@163.com (Y.T.);; 2 Research Center for Chinese Medicine Analysis and Transformation, Beijing University of Chinese Medicine, Beijing 100029, China; 3 Beijing Research Institute of Chinese Medicine, Beijing University of Chinese Medicine, Beijing 100029, China

**Keywords:** *Qishen* granule, effective components, effect–constituent index, in vitro bioassay, pharmacokinetics

## Abstract

**Background**: *Qishen* granule (QSG) is a widely prescribed herbal formula for the treatment of chronic heart failure. The mechanisms of action of QSG have been clarified; however, the effective substances remain unclear. This lack of clarity hinders quality control and the consistency of the clinical efficacy of QSG. **Methods**: In the present study, an integrated strategy for an efficacy- and in vivo exposure-oriented study involving metabolite profiling, molecular docking, in vitro bioassays, and in vivo pharmacokinetics was proposed for investigating the potentially effective components of QSG. **Results**: In total, 101 prototypes/metabolites were preliminarily identified and characterized by UHPLC-Q TOF-MS/MS. Molecular docking of the absorbed constituents with targeted proteins suggested that 49 potential components were highly related to chronic heart failure (CHF). Then, the effectiveness of these potential compounds was verified by the oxygen glucose deprivation/re-oxygenation (OGD/R)-induced H9c2 cell model. As a result, 14 active components were screened, and their median effective concentration (EC_50_) was calculated and utilized to generate the weight coefficient for the bioeffect of each constituent. By exploring the kinetic parameters of the active compounds in a pharmacokinetic study, the exposure levels of these pharmacologically active compounds were determined by area under the curve (AUC_0→∞_) calculations. Finally, by calculating the effect–constituent index (ECI) for each compound, five key active components (cryptochlorogenic acid, chlorogenic acid, isochlorogenic acid C, salvianolic acid B, and neochlorogenic acid), which possess both pharmacological activities and higher exposure levels, were revealed to be the key effective substances of QSG. **Conclusions**: This study is the first to combine pharmacological activities with in vivo exposure for investigating the effective components of QSG. The identification of key active components provides a foundation for improving the quality control of QSG in clinics. The efficacy- and in vivo exposure-oriented integrated method could provide reliable references for other traditional Chinese medicines (TCMs).

## 1. Introduction

*Qishen* granule (QSG), a widely prescribed herbal formula for the treatment of chronic heart failure (CHF), is derived from two popular formulated traditional Chinese medicines (TCMs), which are Simiaoyongan and Zhenwu decoctions, and it is composed of six herbal medicines: Astragali Radix Preparata cum Melle (zhihuangqi, ZHQ), Salvia Miltiorrhiza Radix et Rhizoma (danshen, DS), Aconiti Laterlis Radix Preparata (heishunpian, HSP), Lonicerae Japonicae Flos (jinyinhua, JYH), Scrophulariae Radix (xuanshen, XS), and Glycyrrhizae Radix et Rhizoma Preparata cum Melle (zhigancao, ZGC). The efficacy and safety of QSG in CHF patients have been verified in clinics [1]. The mechanisms of action have also been thoroughly investigated and documented [2,3,4,5,6,7,8]. Quality control based on the content of some vital active compounds is essential to ensure both the safety and efficacy of QSG in clinical applications. However, little research has been carried out to investigate the material’s basis and reveal the effective substances of QSG with respect to CHF. Our previous study identified 213 constituents in QSG, among which the main types were flavonoids, chlorogenic acids, salvianolic acids, tanshinones, iridoid glucoside, alkaloids, phenolic acids, triterpenoid saponins, and phenylpropanoids [9]. While compounds absorbed into the blood are most likely to be responsible for the effects of drugs, investigating the effective substances by exploring the in vivo processes of QSG is an efficient method. There are some reports that focused on pharmacokinetic studies of QSG. The kinetic parameters of the main components were characterized; several compounds, such as glycyrrhizic acid and glycyrrhetic acid, were recognized as the effective components of different kinetic parameters in sham and model rats [10,11,12]. However, these studies focused exclusively on a limited number of compounds selected by the researchers, without comprehensive metabolite profiling of QSG. In addition, the biological activities of the selected compounds were not experimentally validated. Consequently, several vital compounds, including salvianolic acid B from DS, were overlooked despite their high exposure levels in rat plasma, and they exhibited pharmacological activity against CHF.

It is known that TCMs contain numerous ingredients, among which the active compounds exert their effects mostly by being absorbed into the blood and accumulating to an effective concentration [13]. Thus, the investigation of the absorbed components, as well as their exposure levels and efficacies, is of great importance and could provide scientific data for clarifying the effective material basis of TCMs. Pharmacokinetic studies characterize the in vivo exposures of absorbed compounds [13], while in vitro bioassays provide preliminary efficacy evaluations [14]. Usually, exposure is not directly correlated with efficacy. High-exposure compounds may exhibit low activity, while low-exposure compounds can demonstrate high activity. Selecting compounds that possess both substantial exposure and significant pharmacological activity is therefore critical. Nevertheless, an integrated approach combining efficacy and in vivo exposure to identify active constituents of a specific TCM has not yet been reported.

In recent years, the quality marker of the effect–constituent index (ECI) has been proposed for the advancement of the quality control of TCMs [15,16]. This method determines ECI values by measuring the contents of active constituents, with each assigned an effect weight based on its relative pharmacological activities, thereby correlating the results with clinical efficacy and accounting for differential contributions among constituents. Given that TCM quality can be assessed by quantifying both its quality markers and relevant bioeffect indicators, identifying effective constituents may be accomplished by determining the in vivo exposure weighted by the bioactivity coefficient.

**Figure 1:**
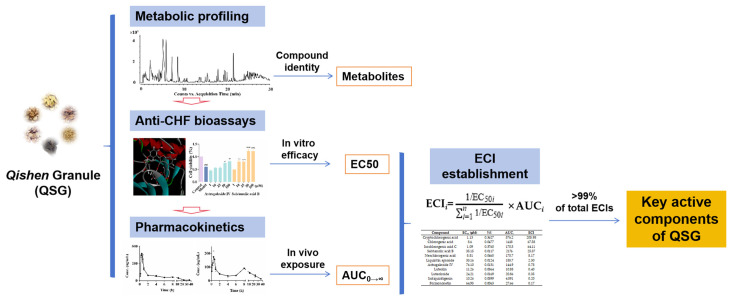
The experimental flow chart of this study.

In this study, an efficacy- and in vivo exposure-oriented integrated method was first developed and utilized for the investigation of the effective components of QSG. Figure 1 shows the experimental flow chart of this study. The ECI for each component was calculated by introducing the bioeffect weight coefficient of median effective concentration (EC_50_) with in vivo exposure; thus, those with both effectiveness and higher exposure levels could be recognized as the effective components of QSG. The results of this study could be helpful for the clarification of the effective substances of QSG, and the identified key active components could also establish a basis for enhancing the quality control of QSG in clinical practice. The efficacy- and in vivo exposure-oriented integrated method for the investigation of effective components could provide reliable references for other TCMs.

## 2. Results

### 2.1. Exploring the Absorbed Components of QSG in Rat Plasma

After oral administration of QSG, a total of 101 components were identified in rat plasma, which included 49 prototypes and 51 metabolites. The detailed identification information is listed in Table 1 and Table 2.

#### 2.1.1. Identification of Prototype Components

A total of 49 prototype components in administered plasma were matched with the established chemical component database of QSG in Metabolite ID software (version B.04.00) [9], which included the chromatographic behaviors and fragmentation patterns of all components identified in QSG. The data of all components, including 16 alkaloids, 11 flavonoids, 6 iridoid glucosides, 5 chlorogenic acids, 5 triterpenoid saponins, 3 tanshinones, 1 salvianolic acid, 1 phenolic acid, and 1 phenylpropanoid, are displayed in Table 1 and Table 2. A pair of isomers, compounds **38** and **50**, were selected as the examples for the stepwise elucidations of the molecular structures. Liquiritin and isoliquiritin are two important compounds previously identified in QSG [9]; thus their MS information such as molecular formulas and retention times as well as their MS2 spectrum was imported into Metabolite ID for their metabolite identification. There were two isomers screened with precursor ions of *m*/*z* 417.1191 in QSG-administered rat plasma, and diagnostic fragment ions at *m*/*z* 135.008 and *m*/*z* 119.0502 generated from Retro-Diels–Alder (RDA) cleavage of Ring C confirmed that they were flavanones and/or chalcones [17]; they were further identified as liquiritin and isoliquiritin by comparing the MS information with that of the standards imported in Metabolite ID (Figure S1).

#### 2.1.2. Identification of Metabolites

Based on the metabolic prediction and screening function of Metabolite ID, a total of 52 metabolites were identified, which included 21 flavonoid metabolites, 16 phenolic acid metabolites, 8 tanshinone metabolites, 5 alkaloid metabolites, and 2 salvianolic acid metabolites. The metabolic phase II reactions were mainly glucuronide and sulfate conjugation, phase I reactions mainly consisted of hydrogenation and hydrolysis, and phase I+phase II conjugations such as glucuronidation+hydrogenation were also detected in this study.

A total of 21 flavonoid metabolites including 17 phase II and 4 phase I+II conjugation metabolites were identified. Fifteen compounds (**18**, **20**, **24**, **29**, **31**, **33**, **36**, **41**, **48**, **52**, **55**–**58**, **60**) were characterized as glucuronide conjugates due to the neutral loss of 176.0321 Da. Most of these compounds were deduced as isomers because of their identical molecular weights as well as similar fragment pathways; thus glucuronide should be bonded to different substituent positions of one compound or the same substituent position of different isomers. In our study, the structures of most compounds were characterized based on MS2 fragmentation patterns and comparisons with literature data, enabling their use in subsequent molecular docking experiments. For example, **18** (t_R_ = 5.86 min) and **29** (t_R_ = 8.70 min) displayed [M−H]^−^ ions at *m*/*z* 593.1512 and 593.1501, with fragment ions such as *m*/*z* 417.1189 and 255.0671 indicating that they are glucuronidation metabolites of liquiritin and isoliquiritin, and conjugated positions should be 7-OH. By comparing the ClogP values of **18** and **29**, they were deduced as liquiritin-7-O-GluA (ClogP, −1.22) and isoliquiritin-7-O-GluA (ClogP, −0.69) [18,19,20]. Similarly, **31**, **33**, and **55** were confirmed as glucuronidation metabolites of liquiritigenin and isoliquiritigenin, and **31** and **33** were deduced as liquiritigenin-7-*O*-glucuronide (ClogP, 0.27) and liquiritigenin-4′-*O*-glucuronide (ClogP, 0.56) [18,19,20]. However, the substituted position of **55** could not be accurately determined due to the presence of two hydroxyl substituents in the isoliquiritigenin structure. In addition, **27** was identified as a glucuronidation+sulfation metabolite of liquiritigenin, and **43** was a sulfation metabolite of calycosin. Four compounds (**34**, **42**, **46**, **54**) were identified as phase I+II conjugation metabolites; among them, **54** was identified as a hydrogenation+glucuronidation metabolite of liquiritigenin or isoliquiritigenin [18,19,20]. The fragment ion was at *m*/*z* 257.0818, which was 2 Da more than liquiritigenin or isoliquiritigenin, indicating that it is metabolized by a hydrogenation reaction. To determine whether metabolite 54 originated from liquiritigenin or isoliquiritigenin, we compared the behaviors of compounds **54** and **55**. Compound **55** (t_R_ = 13.31 min) was identified as the glucuronide conjugate of isoliquiritigenin. A hydrogenated and glucuronidated derivative of isoliquiritigenin would be expected to display greater hydrophobicity. The shorter retention time of compound **54** (t_R_ = 12.93 min), however, indicated that it more likely arises from liquiritigenin through hydrogenation and subsequent glucuronidation. Precursor ions of *m*/*z* 513.071 [M−H]^−^ were found for **34**, **42**, and **46**, which were 80 Da higher than **54**, indicating that they undergo another sulfation reaction. Based on the ClogP values of the three metabolites, **34** was identified as a hydrogenation+glucuronidation+sulfation metabolite of liquiritigenin, and **42** and **46** were isoliquiritigenin+H_2_-7-*O*-GluA-4′-SO_3_ and isoliquiritigenin+H_2_-4′-*O*-GluA-7-SO_3_. Moreover, **41**, **48**, and **56** were preliminarily identified as glucuronidation metabolites of naringenin by Metabolite ID. According to the identification results of the chemical components of QSG [9], the three metabolites were position isomers distinguished by different conjugating sites on the three hydroxyl groups of naringenin; according to their ClogP values, they were identified as naringenin-5-*O*-GluA (ClogP, −0.05), naringenin-7-*O*-GluA (ClogP, 0.41) and naringenin-4′-*O*-GluA (ClogP, 0.47). Similarly, **20**, **24**, **36**, **52**, and **57** were identified as glucuronidation metabolites of liquiritin apioside, calycosin-7-*O*-D-glucoside, calycosin, formononetin, and 3′-methoxy-luteolin. **43** was a sulfation metabolite of calycosin.

A total of 16 phenolic acid metabolites including 11 phase II and 5 phase I metabolites were identified. **2**–**5**, **7**, **15**, **21**, and **28** were identified as sulfonic acid conjugates due to the neutral loss of SO_3_ residues (79.9568 Da). With their identical precursor ions at *m*/*z* 232.9771 and similar fragment ions of **2** and **4** indicating that they are isomers, they were further confirmed as protocatechuic acid-3-*O*-SO_3_ and protocatechuic acid-4-*O*-SO_3_ with substitution positions at the two hydroxyl sites. **5** and **7** were confirmed to be sulfation metabolites of vanillic acid. **3** and **15** were recognized as sulfation metabolites of danshensu and caffeic acid. **11**, **17**, and **19** were metabolites of glucuronidation, confirmed by the neutral loss of 176.0321 Da. **17** and **19**, two isomers, were identified as (*E*)-ferulic acid-4-*O*-GluA and (*Z*)-ferulic acid-4-*O*-GluA based on their ClogP values. Similarly, **21** and **28** were characterized as (*E*)-ferulic acid-4-*O*-SO_3_ and (*Z*)-ferulic acid-4-*O*-SO_3_. The fragment ions of **11**, such as *m*/*z* 179.0357 and 135.0450, confirmed that it is the glucuronidation metabolite of caffeic acid.

**91**, **93**, **94**, **96**, and **98**–**101** were characterized as metabolites of tanshinone IIA, and the metabolized reactions mainly involve hydration, hydrogenation, and demethylation. For example, **94** and **101** were screened out as the hydration+hydrogenation metabolites of tanshinone IIA, and the fragment ion at *m*/*z* 297.1494, which is 2 Da higher than tanshinone IIA, was generated by the neutral loss of H_2_O; **94** and **101** were thus confirmed as the hydration+hydrogenation product of tanshinone IIA. **96**, which was observed at *m*/*z* 301.1439 [M+H]^+^, is 15 Da lower than **94** and **101**, with similar fragmentation patterns indicating that they are isomers of the hydration+hydrogenation+demethylation products of tanshinone IIA. These three metabolites (**94**, **96** and **101**) were first reported in this study, and they were identified as potential new ones; however, detailed structure information still needs more research. Moreover, **93** and **100** were screened as hydrogenation metabolites of tanshinone IIA, **98** and **99** as hydroxylation+demethylation and hydroxylation metabolites of tanshinone IIA, and **91** as a hydration metabolite of tanshinone IIA.

Five alkaloid-related metabolites (**71**, **72**, **77**, **79**) were identified by Metabolite ID, all of which were isomers of the prototype components in QSG, though their specific structures could not be determined yet. Two metabolite isomers related to rosmarinic acid (**45** and **51**) were identified, which were the methylation metabolites of rosmarinic acid with different reaction positions.

### 2.2. Anti-CHF Screening and Experimental Verification

#### 2.2.1. Molecular Docking Assessments

**Figure 2:**
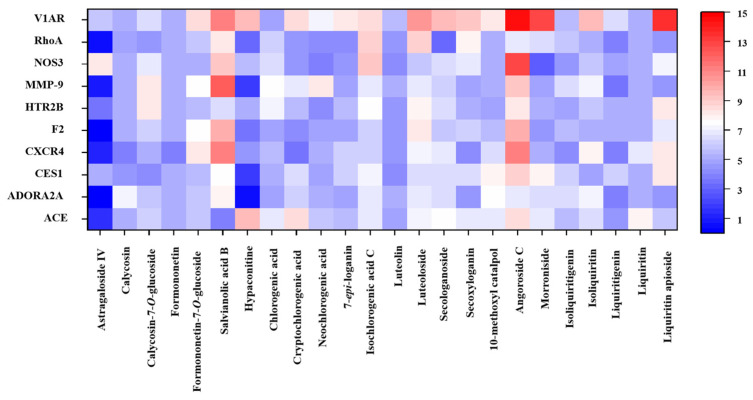
Heatmap of molecular docking results for 24 components with 10 core targets.

Among the 101 identified components, the structures of 22 could not be accurately determined. The remaining 79 small molecules (Table S1) were therefore eventually utilized for subsequent molecular docking assessment with 36 target proteins (Table S2). As a result, a total of 49 components, including 24 prototypes and 25 metabolites, with a total score ≥ 7 for at least one target protein were screened. Tables S3 and S4 show the molecular docking results of 49 components with 24 target proteins. Twenty-five metabolites primarily originated from these 24 prototypes; these prototype components were therefore identified as potential active constituents closely associated with CHF. Figure 2 displays the associations of the 24 prototypes with the top 10 target proteins. Five components, namely calycosin, calycosin-7-*O*-glucoside, formononetin, formononetin-7-*O*-glucoside, and astragaloside IV, are derived from ZHQ. Salvianolic acid B was derived from DS, and hypaconitine was derived from HSP. Nine components including luteolin, luteoloside, neochlorogenic acid, cryptochlorogenic acid, chlorogenic acid, isochlorogenic acid C, 7-*epi*-loganin, secoxyloganin, and secologanoside were derived from JYH; 10-methoxyl catalpol, morroniside, and angoroside C were three components derived from XS. The five components of isoliquiritigenin, liquiritigenin, liquiritin, isoliquiritin, and liquiritin apioside were derived from ZGC.

**Figure 3:**
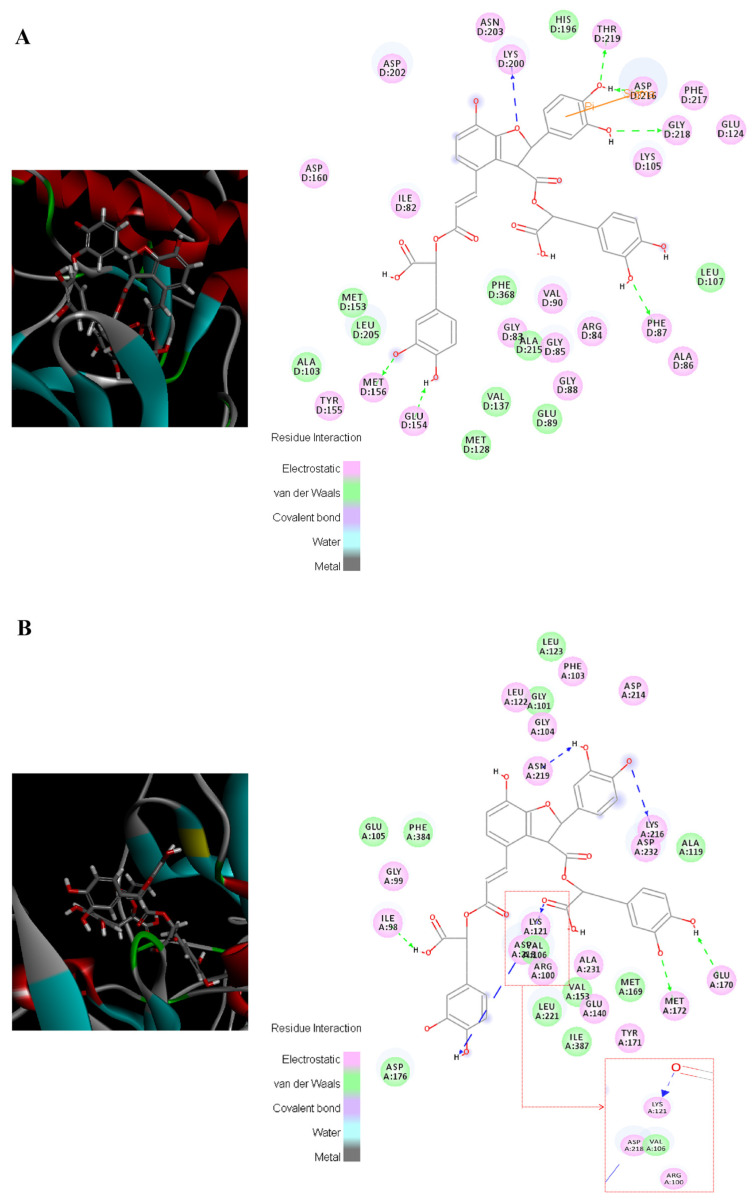
Visualization of the binding of salvianolic acid B with ROCK1 and ROCK2. (**A**) ROCK1; (**B**) ROCK 2.

Here, the bindings between salvianolic acid B and the core proteins related to myocardial fibrosis are introduced as an example. ROCK1 and ROCK2 were the key molecules in the RhoA/ROCK signaling pathway; as shown in Figure 3, salvianolic acid B could tightly bind to ROCK1 and ROCK2 (Total score > 10), and electrostatic, van der Waals, and covalent bond interactions were mainly formed between the amino acid residues of the target proteins and the nucleus of salvianolic acid B. The complexes of salvianolic acid B with ROCK1 and ROCK2 were stabilized by interactions with amino acid residues LYS D:200, THR D:219, ASP D:216, GLY D:218, PHE D:87, GLU D:154, MET D:156 and ASN D:219, LYS A:216, GLU A:170 MET A:172, LYS A:121, ASP A: 218, and ILE A:98, as well as by hydrogen bonds. Salvianolic acid B formed one pi–sigma interaction with ASP D: 216 in ROCK 1. Docking results of salvianolic acid B and other components with target proteins are listed in Tables S3 and S4. It is worth mentioning that salvianolic acid B is present in the highest concentrations of all components in QSG as well as in the QSG-administered plasma among all target compounds (Table S5); thus it is considered the key active compound in QSG, which is worthy of in-depth research.

#### 2.2.2. Experimental Verification by Oxygen Glucose Deprivation/Re-Oxygenation (OGD/R)-Induced H9c2 Cells

Firstly, CCK-8 results demonstrated that most components exhibited no cytotoxicity toward H9c2 cells (Table S6). Notably, treatment with cryptochlorogenic acid, isochlorogenic acid C, and salvianolic acid B significantly enhanced cell viabilities compared to the control group, indicating their potent pro-proliferative effects. In the OGD/R model, H9c2 cell viability showed a significant decline (*p* < 0.001), which confirmed the successful induction of cell injury and apoptosis.

**Figure 4:**
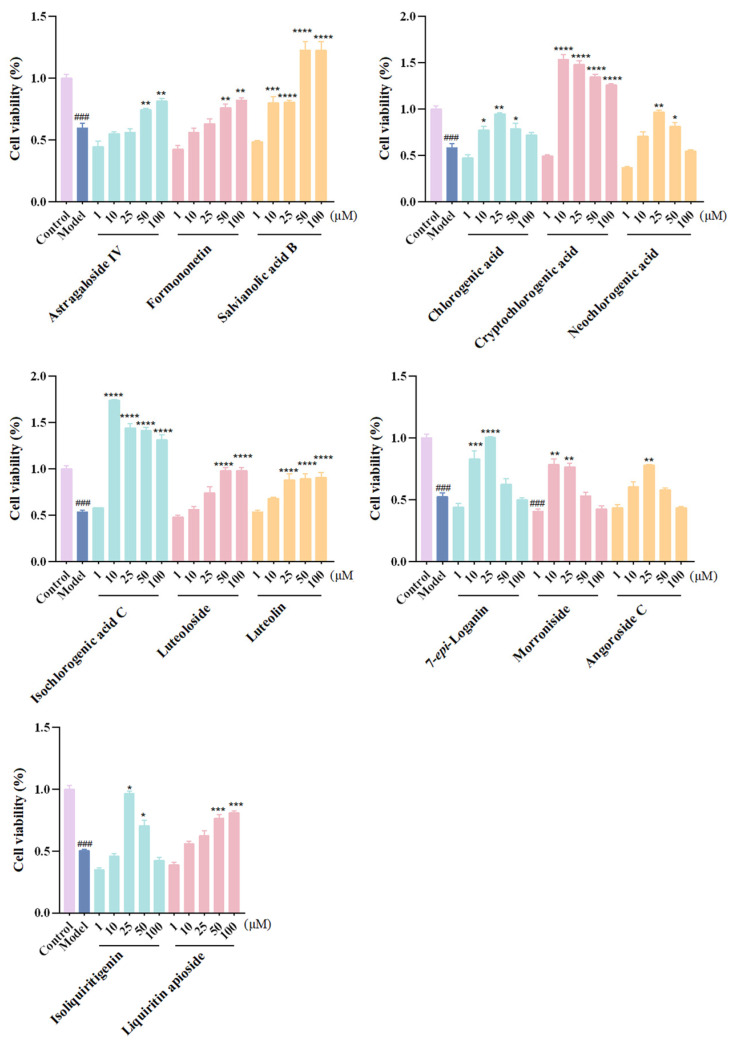
Anti-CHF effects of active ingredients on OGD/R-induced H9c2 cells. Data are expressed as the mean ± SD. *n* = 6; ###, *p* < 0.001; compared with the control group. ****, *p* < 0.0001; ***, *p* < 0.001; **, *p* < 0.01; *, *p* < 0.05; compared with the model group.

Afterward, the 24 screened potentially effective components toward CHF were verified by assessing the protective effects in alleviating OGD/R injury on rat myocardial H9c2 cells. Results suggested that 14 analytes, which were cryptochlorogenic acid, isochlorogenic acid C, salvianolic acid B, luteolin, loganin, morroniside, isoliquiritigenin, chlorogenic acid, neochlorogenic acid, angoroside C, luteoloside, liquiritin apioside, formononetin, and astragaloside IV, could significantly improve H9c2 cell survival rates at different concentrations, as shown in Figure 4 (the pharmacological activities for the remaining 10 compounds are shown in Figure S2). Among them, astragaloside IV and formononetin, which originated from the monarch drug ZHQ, exhibited significant protective effects at 50 and 100 μM. Salvianolic acid B in ministerial drug DS, which showed the highest content in vitro and in vivo, exerted strong cardioprotective activity, thus highlighting its crucial role in the therapeutic effects of QSG. The “jiedu” principle embodied by adjunct drugs JYH and XS distinguishes QSG from other cardiovascular formulas [21]. As could be seen in the results, chlorogenic acid, cryptochlorogenic acid, neochlorogenic acid, isochlorogenic acid C, luteolin, luteoloside, and 7-*epi*-loganin in JYH, as well as angoroside C and morroniside in XS, significantly enhanced cardiomyocyte survival, which validated their efficacy-enhancing roles in CHF treatment. Moreover, isoliquiritigenin and liquiritin apioside, two active constituents, originated from the courier drug ZGC.

It is reported that astragaloside IV could activate PPARα, shifting energy metabolism from glycolysis toward fatty acid β-oxidation and promoting angiogenesis [22,23]. Formononetin reduces myocardial ischemia/reperfusion injury by alleviating thrombosis and inflammation [24]. Salvianolic acid B markedly preserves left ventricular structure and cardiac function through its action on matrix metalloproteinase-9 [25]. Chlorogenic acid protects cardiomyocytes from TNF-α-induced injury by targeting NF-κB and JNK signaling pathways [26,27]. Cryptochlorogenic acid enhances hemodynamic function and ameliorates cellular morphology in myocardial tissues subjected to ischemia/reperfusion [28]. Both neochlorogenic acid and isochlorogenic acid C exhibit protective effects on H9c2 cells against ISO-induced injury [29]. Luteolin safeguards myocardial cells by suppressing apoptosis and oxidative stress [30]. Luteoloside mitigates damage to myocardial cells caused by hypoxia/reoxygenation [31]. 7-*epi*-loganin inhibits angiotensin II-induced cardiac hypertrophy [32]. Angoroside C improves ventricular remodeling in rats under pressure overload [33]. Morroniside promotes cardiac repair and exerts cardioprotective effects following myocardial infarction in adult rats [34]. Isoliquiritigenin protects against heart failure in mice via anti-inflammatory and anti-remodeling mechanisms [35].

All 14 active components, representing either quality control marker compounds or high-content constituents in their respective herbs, demonstrated protective effects against OGD/R injury and should be the material basis for the anti-CHF efficacy of QSG.

### 2.3. In Vivo Exposure Determination of Screened Active Components by Pharmacokinetic Study

Previous studies suggested that of the 14 active compounds, 7-*epi*-loganin, morroniside, and angoroside C exhibit relatively lower exposure levels in vivo (Table S5); thus the other 11 components were applied for the pharmacokinetic study to explore their exposures as well as other kinetic parameters.

#### 2.3.1. Method Validation

**Figure 5:**
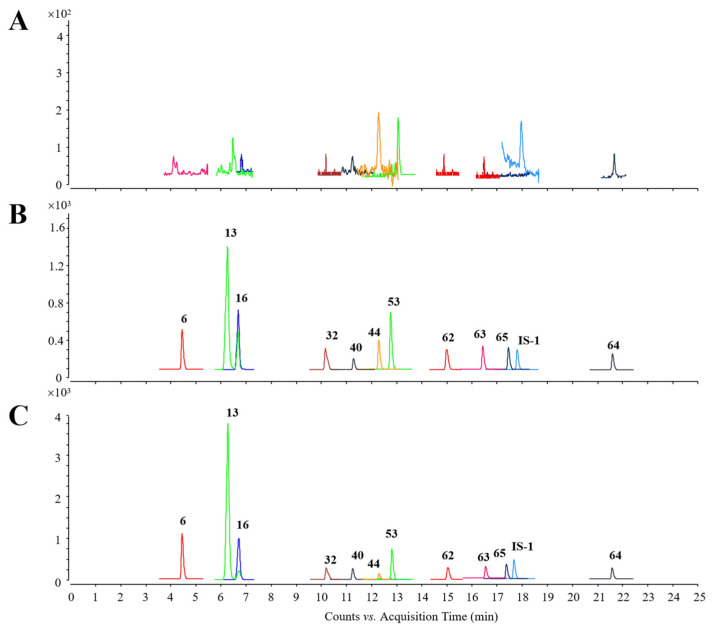
Representative chromatograms. (**A**) Blank plasma sample. (**B**) QC plasma sample. (**C**) Plasma sample obtained 30 min after QSG treatment.

Figure 5 displays the representative chromatograms of the blank plasma sample, quality control (QC) plasma sample, and plasma sample obtained 30 min after QSG treatment, with no apparent interference from the endogenous matrix interfering with the quantitation of both the analytes and internal standard (IS). Compound parameters, such as ion transitions, fragmentors, and collision energies (CEs), are summarized in Table S7. Calibration curves are presented in Table S8, and the correlation coefficients (R^2^) were all above 0.9973. Limits of detection (LODs) for 11 analytes ranged from 0.01 to 1.0 ng/mL. As shown in Table S9, the relative standard deviation (RSD) of intra-day and inter-day precision varied within the ranges of 1.02–13.68% and 2.59–12.02%, respectively. The accuracy ranged from 85.10 to 114.70% and 85.32 to 114.74%. Results of stability tests showed that the RSDs of all analytes were within 15% and the accuracy data ranged from 85.10% to 114.83% (Table S10), indicating good stabilities. As illustrated in Table S11, matrix effects ranged from 85.12% to 108.33%, and the recoveries of these 11 analytes ranged from 85.81% to 114.73%.

#### 2.3.2. Pharmacokinetic Analysis

**Figure 6:**
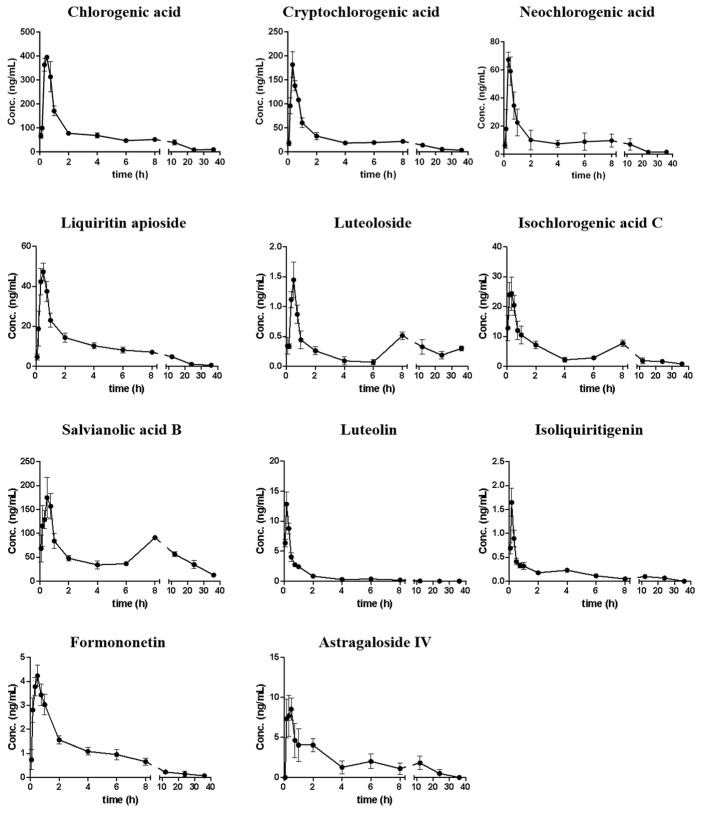
Mean (SD, *n* = 6) plasma concentration–time curves of 11 analytes after oral administration of QSG in rats.

The developed method was applied for pharmacokinetic investigations of 11 analytes in rats after oral administration of 7 g/kg QSG. Figure 6 shows the mean plasma concentration–time curves of all analytes, with the key parameters such as area under the curve (AUC_0→∞_) summarized in Table 3.

It was observed that 11 compounds could be detected in plasma within 5 min, and most compounds reach their C_max_ within 40 min, which indicated that these compounds could be rapidly absorbed into the circulation system. In addition, the mean residence time (MRT) of each analyte ranged from 1.839 to 16.01 h, with most compounds retained for more than 5 h. Among them, the MRTs of luteoloside and salvianolic acid B were more than 10 h, indicating that they were retained in the circulation system for a relatively long time; however, most compounds could be eliminated within 36 h. Double peaks were observed for luteoloside, isoliquiritigenin, isochlorogenic acid C, salvianolic acid B, and astragaloside IV. They reached the first peak at 10–30 min, and then reached the second peak at 4–12 h, with the concentrations of their second peak lower than those of the first peak, which might be attributed to the hepatic and intestinal circulations. In addition, the second peak for compounds like isoliquiritigenin might also be due to the glycolysis of liquiritin apioside, liquiritin, and isoliquiritin existing in QSG [36].

AUC_0→∞_ results suggested that salvianolic acid B exhibited the highest in vivo exposure level of 2176 ± 842.3 ng h/mL, accounting for 43.48% of the total exposures of all analytes. The exposure levels of four chlorogenic acid compounds (chlorogenic acid, neochlorogenic acid, cryptochlorogenic acid, and isochlorogenic acid C) accounted for another 46.76%. By comparing the exposure level (AUC_0→∞_) with the dosage of each compound, the oral relative bioavailability was obtained (Table S12). Among them, astragaloside IV had relatively higher absorption efficiency. It is worth noting that although salvianolic acid B had the highest content in QSG and the highest exposure level in vivo, the bioavailability was much lower than most compounds, which might be related to the physicochemical properties and/or the oversaturation condition for salvianolic acid B in vivo. Most flavonoid aglycones had a higher absorption rate than flavonoid glycosides, which might have resulted from the hydrolysis of glycosides into their aglycones in vivo. The four chlorogenic acid analogs exhibited markedly different absorption efficiencies. Notably, neochlorogenic acid and cryptochlorogenic acid demonstrated relatively higher absorption rates, whereas isochlorogenic acid C and chlorogenic acid were less readily absorbed. These results suggested that neochlorogenic acid and cryptochlorogenic acid might represent the dominant conformers in vivo. The other two compounds may undergo hydrolysis or isomerization to be converted into these two analogs. Overall, the results are helpful in understanding the kinetic parameters of active compounds of QSG and provide scientific data for ECI calculation.

### 2.4. Exploration of the Effective Components by the Efficacy- and In Vivo Exposure-Oriented Integrated Method

According to the in vitro bioeffect assay, the EC_50_ values for 11 constituents were calculated and are presented in Table 4; the ranking of the 11 compounds is clearly different from the AUC_0→∞_ ranking. It is difficult to identify the key effective components in the anti-CHF activities of QSG according to the distinct bioeffects and in vivo exposures.

To address this challenge, we developed an efficacy- and in vivo exposure-oriented integrated strategy to identify the key active constituents of QSG, using a comparison of their ECIs. The ECI for each constituent was calculated as the product of its in vivo exposure (AUC_0→∞_) and a weighting factor (*Wi*) derived from its bioactivity (EC_50_). Since EC_50_ values are inversely related to compound activity, each constituent was assigned a normalized weight using the reciprocal of its EC_50_. We then applied Equation (1) to compute *Wi* from the EC_50_ values of the 11 compounds, and subsequently used Equation (2) to determine their respective ECIs by incorporating both *Wi* and AUC_0→∞_.

According to the results shown in Table 4, the key effective components could be readily recognized by their individual indicator of the ECI, which ranked active constituents by correlating bioeffects and dealing with the in vivo exposure differences. For example, salvianolic acid B exhibited the highest exposure level with a moderate bioeffect, while isocholorgenic acid C possessed lower exposure but the strongest bioeffect, and their contributions to the effective material basis were ranked as isocholorgenic acid C > salvianolic acid B by their ECIs. Consequently, cryptochlorogenic acid, chlorogenic acid, isochlorogenic acid C, neochlorogenic acid, and salvianolic acid B were preliminarily identified as the key active components of QSG, with each exhibiting an ECI > 5, collectively accounting for approximately 99% of the total ECIs.

## 3. Discussion

In this study, we developed and applied an integrated strategy oriented toward efficacy and in vivo exposure to identify effective constituents of QSG. Identifying effective compounds requires consideration of both high pharmacological activities and adequate in vivo exposures. Our results indicated, however, that exposure levels are not directly correlated with efficacies: high-exposure compounds such as salvianolic acid B showed relatively low activity, whereas isochlorogenic acid C showed a strong effect with lower exposure. To pinpoint key compounds combining substantial exposures with high pharmacological activities, we introduced the multi-criteria evaluation metric ECI to integrate both exposure and efficacy contributions. By incorporating bioactivities as weighting factors for in vivo exposure levels, five kinetic active components demonstrating both anti-CHF effects and elevated in vivo exposure levels were identified as key effective constituents of QSG. Consequently, the content of these active ingredients can be controlled to ensure the efficacy and safety of QSG, thereby enhancing the quality control in clinical applications.

To the best of our knowledge, this is the first study to combine the in vivo exposures of the constituents (considering the in vivo kinetic parameters) with their bioeffects for the exploration of the effective components in a TCM. However, there are also some limitations in our study; for instance, the bioeffects of the absorbed components were only judged by one single-cell model, and the ECI threshold for key effective components needs in-depth studies.

## 4. Materials and Methods

### 4.1. Chemicals and Materials

Chlorogenic acid, cryptochlorogenic acid, neochlorogenic acid, isochlorogenic acid C, calycosin-7-*O*-D-glucoside, formononetin-7-*O*-D-glucoside, salvianolic acid B, astragaloside II, astragaloside IV, morroniside, sweroside, angoroside C, luteolin, glycyrrhetic acid, 7-*epi*-loganin, secologanoside, secoxyloganin, and 10-methoxyl catalpol were obtained from Chengdu Herb purify Co., Ltd. (Chengdu, China). Formononetin, luteoloside, calycosin, liquiritin apioside, liquiritin, liquiritigenin, isoliquiritin, isoliquiritigenin, benzoylmesaconine, benzoylhypacoitine, hypaconitine, dihydrotanshinone I, and glycyrrhizic acid were purchased from Shanghai Standard Biotech Co. Ltd. (Shanghai, China). Benzoylaconine and tanshinone IIA were obtained from National Institutes for Food and Drug Control (Beijing, China). The purities of these standards were all above 98%.

LC-MS-grade acetonitrile, methanol, and formic acid were purchased from Fisher-Scientific (Fair Lawn, NJ, USA). Ultrapure water was prepared by a Milli-Q system (Millipore, Bedford, MA, USA).

QSG was prepared in our group following a previous protocol [9].

### 4.2. Instrument and Conditions

#### 4.2.1. UHPLC-Q TOF-MS Conditions

An Agilent Q-TOF 6550 iFunnel mass spectrometer (Agilent Technologies, Santa Clara, CA, USA) was operated using the following parameters according to our previous study [9]: the fragmentor and nebulizer were set at 380 V and 35 psig, temperatures of drying gas and sheath gas were 150 °C and 275 °C, flow rates of drying gas and sheath gas were 16 L/min and 11 L/min, capillary voltages were set as 4000 V (+) and 3500 V (−), the full scan mass range was *m*/*z* 100–1200, and PIS-MS/MS, following the previous protocols, was utilized for MS2 data acquisition. UHPLC was performed on an Agilent 1290 Infinity UHPLC system (Agilent Technologies, CA, USA). The separation of metabolites was achieved on an ACQUITY UPLC HSS T3 column (100 mm × 2.1 mm, 1.8 μm, Waters, Wexford, Ireland); 0.1% formic acid in water (A) and 0.1% formic acid in acetonitrile (B) were used as the mobile phases, the flow rate was set as 0.4 mL/min, the column oven was maintained at 35 °C, and the gradient procedure was as follows: 0–10 min, 5–23% B; 10–15 min, 23–30% B; 15–20 min, 30–60% B; 20–25 min, 60–95% B; and 25–30 min, 95% B. The injection volume was set at 5 μL.

#### 4.2.2. HPLC-QQQ-MS Conditions

An Agilent 6470 QQQ mass spectrometer (Agilent Technologies, Santa Clara, CA, USA) via an electrospray ionization (ESI) interface was employed for analyte determination, with dynamic MRM mode in negative mode utilized. Ion source parameters were set as follows: temperatures of drying gas and sheath gas were 300 °C and 300 °C, flow rates of drying gas and sheath gas were 7 L/min and 11 L/min, the nebulizer was set at 35 psig, capillary voltage was 3500 V (−), and nozzle voltage was 2000 V (−). As for the HPLC domain, an Agilent 1260 Infinity HPLC system was utilized. Chromatographic separations were conducted on a ZORBAX Eclipse Plus C18 column (3.0 × 50 mm, 1.8 μm, Agilent Technologies, California, USA). The mobile phase was composed of 0.1% aqueous FA (A, (*v*/*v*)) and 0.1% formic acid methanol (B), the flow rate was set at 0.5 mL/min, and the column oven was maintained at 40 °C. The gradient elution was programmed as follows: 0–5 min, 5–25% B; 5–15 min, 25–55% B; 15–20 min, 55–85% B; 20–22 min, 85–95% B; and 22–25 min, 95% B. The injection volume was set at 10 μL.

### 4.3. Experimental Animals

Male Sprague Dawley rats (200 ± 20 g) were bought from Vital River Laboratory Animal Technology Co., Ltd. {(SCXK (Jing) 2021~0006, Beijing, China}. The rats were fed under a humidity of 50–70% and temperature of 22–25 °C with a light–dark cycle of 12 h. All animals were fasted overnight with free access to water prior to drug administration.

### 4.4. Identification of the Absorbed Components of QSG in Rat Plasma

#### 4.4.1. Preparations of Standard Solutions and Plasma Samples

Stock standard solutions of 25 compounds were mixed and diluted to 500 ng/mL for compound confirmation. For metabolic profiling, 10 rats were randomly divided into the QSG group (*n* = 5) and control group (*n* = 5), and after a single oral administration of 7 g/kg of QSG or water, blood was collected from each rat at 0.5, 1, 4, 8, and 12 h. Afterward, blood samples of each time point were mixed in equal amounts, and 9 mL of acetonitrile was immediately added to 3 mL of the pooled plasma for protein precipitation. After 5 min of centrifugation (13,201× *g*, 4 °C), the supernatant was moved to a new vial for concentration, and the dried residue was reconstituted with 150 μL 50% aqueous acetonitrile (*v*/*v*) and centrifuged for another 5 min (13,201× *g*, 4 °C).

#### 4.4.2. Metabolite Identification

A database of 213 chemical components including their MS and MS2 information was established for QSG based on results previously reported [9]; the database was further imported into Metabolite ID so as to predict possible metabolites of QSG in vivo. Then, the acquired data files of administered plasma were introduced into Metabolite ID, and parameters such as error tolerance (±10 ppm) and peak abundance threshold (>10^3^) were set. Meanwhile, the data files of blank plasma were also imported into Metabolite ID; thus components detected in blank plasma samples could be automatically excluded, making the analysis results more reliable. As a result, compounds that met the requirements were extracted and highlighted, and MS/MS data of all targeted metabolites were further compared with their prototypes for structure identities.

### 4.5. Screening of the Anti-CHF Active Components

#### 4.5.1. Molecular Docking Screening

To evaluate the binding affinity of the absorbed components to CHF-related target proteins, molecular docking studies were performed by using SYBYL-X software (version 2.0). The three-dimensional structures of the absorbed components were constructed and energy-minimized. The target proteins were acquired by combining the reported anti-CHF targets of QSG [3,4,5,6,7,37,38,39] and targets in the Therapeutic Target Database (https://db.idrblab.net/ttd/, accessed on 9 September 2023) of marketed drugs, and three-dimensional structures of these targets were acquired from the Protein Data Bank (https://www.rcsb.org/, accessed on 9 September 2023) database. Docking was performed using the Surflex-Dock Geom (SFXC) mode. The target protein was prepared by adding hydrogen atoms, followed by the removal of ligands, water molecules, and heteroatoms. The therapeutic effects were assessed by the docking scores; the higher the total score, the more stable the binding conformation. Typically, a total score ≥ 7 often denotes great docking [40]; thus molecules with docking scores above the threshold were considered to be potential effective components.

#### 4.5.2. Bioassay Screening

Rat myocardial H9c2 cells (purchased from China Infrastructure of Cell Line Resources, Institute of Basic Medical Sciences, Chinese Academy of Medical Sciences) were cultured in DMEM supplemented with 10% fetal bovine serum (FBS), Penicillin (100 U/mL), and Streptomycin (100 μg/mL) at 37 °C under a humidified atmosphere of 5% CO_2_ and 95% air. For OGD/R treatment, H9c2 cells were cultured in glucose-free DMEM under hypoxic conditions (95%N_2_ + 5%CO_2_) for 4 h at 37 °C followed by complete medium under normal conditions with air (95%) and CO_2_ (5%) for 2 h. A control group was maintained under normal culture conditions (with glucose-containing DMEM and 5% CO_2_) without undergoing OGD/R treatment. CCK8 was used to assess cell viability. After treatment, H9c2 cells were seeded in 96-well plates, with each well supplemented with 10 μL of CCK-8 solution and then incubated for 1 h. Finally, the absorbance at 450 nm was detected by a microplate reader (Model INFINITE 200 PRO, TECAN, Grodig, Austria).

When evaluating the cytotoxicities of the absorbed compounds, the H9c2 cells were seeded in 96-well plates at a density of 1 × 10^4^ cells/well; when cells reached 80% confluence, they were treated with 25 and 50 μM of the 24 analytes to screen the non-toxic concentration ranges. Afterward, the protective effects were verified by assessing the cell viability when treating with different concentrations (1, 10, 25, 50, and 100 μM) of the analytes prior to OGD/R treatment.

### 4.6. In Vivo Exposure Determination of Screened Active Components by Pharmacokinetic Study

#### 4.6.1. Preparations of Standard Solutions and Plasma Samples

Working mixed standard solutions were made by diluting the stock solutions to a series of concentrations. QC samples were three concentration levels of calibration samples. Afterward, 10 μL of each working solution or QC solution was spiked into 100 μL blank plasma to generate calibration samples or QC samples with desired concentrations.

#### 4.6.2. Method Development

Several columns and mobile phases were assayed to achieve satisfactory separations. Different biosample pretreatment methods, such as protein precipitation and liquid–liquid extraction, were also compared to enhance the recoveries of these analytes.

The developed method was fully carried out according to the FDA guidelines [41]. The calibration curve was plotted by the peak area ratio of analyte/IS against the theoretical concentration. Linearity for each analyte was assessed with over seven concentrations. Concentrations at a signal-to-noise ratio (S/N) of about 3 were determined as the LODs. The selectivity of the method was validated by comparing the blank plasma, QC plasma, and QSG-administered plasma samples. Precision and accuracy were examined by measuring six replicate QC samples on one day and two replicate QC samples on three consecutive days. The RSD should be within 15%. Stabilities such as in the short term, in the long term, and under three cycles of freeze–thaw assays were assessed by storing the QC samples at room temperature (25 °C) for 24 h, −80 °C for 30 days, −80 °C for 24 h and thawing at 25 °C for three cycles. The stability was acceptable when that of all analytes ranged from 85% to 115%, with the RSD not exceeding 15%. The matrix effects were evaluated by comparing the post-extracted samples with those of standard solutions. The recoveries were evaluated by calculating the peak areas between QC samples and spiked-after-extraction samples.

#### 4.6.3. Pharmacokinetic Study

For the pharmacokinetic study, each rat (*n* = 6) received a single oral administration of 7 g/kg of QSG, and the collection time points were 0 (pre-dose), 0.03, 0.08, 0.17, 0.33, 0.5, 0.75, 1, 2, 4, 6, 8, 12, 24, and 36 h. The blood samples were immediately centrifugated at 1467× *g* (4 °C) for 20 min, and the homogenate supernatants were collected for the following analysis. Protein precipitation was carried out for each time point for plasma samples, calibration samples, or QC samples by introducing four volumes of acetonitrile containing 400 ng/mL of icariin. Then, 10 μL 10% aqueous ascorbic acid (*v*/*v*) was spiked into each sample to prevent the oxidation of phenolic compounds. The precipitates were removed by 10 min of centrifugation (13,201× *g*, 4 °C). An aliquot (450 μL) of the supernatant was transferred and dried under nitrogen vacuum. The residues were reconstituted with 100 μL of 50% aqueous methanol (*v*/*v*) and centrifuged for another 5 min at 13,201× *g* (4 °C).

The pharmacokinetic parameters were calculated by DAS software (version 2.0) for a non-compartmental analysis.

### 4.7. ECI Establishment

It is known that the greater the EC_50_ value, the lower the pharmacological activity. Thus, we calculated the protective effect coefficient for each constituent using the normalization reciprocal value of EC_50_ in Equation (1):

(1) $$W_{i}=\frac{1/{\mathrm{EC}}_{50i}}{\sum_{i=1}^{n}1/{\mathrm{EC}}_{50i}}$$

*Wi* refers to the weight coefficient for the bioeffect of each constituent in Table 4. EC_50*i*_ is the EC_50_ of constituent *i*. *n* is the number of constituents. By inputting EC_50_ values of each constituent to the above equation, the ECI models of anti-CHF activity for each active compound can be derived, as shown in Equation (2):

ECI*_i_* = *W_i_* × AUC*_i_* (2)

In the equations shown above, ECI*_i_* represents the predicted protective effect in alleviating OGD/R injury on H9c2 cells. AUC*_i_* is the exposure of each active component in QSG.

## 5. Conclusions

In the present study, an efficacy- and in vivo exposure-oriented integrated strategy of metabolite profiling, molecular docking, in vitro bioassays, and in vivo pharmacokinetics was established to systematically characterize the absorbed components as well as reveal the key effective components of QSG. Five kinetic active components (cryptochlorogenic acid, chlorogenic acid, isochlorogenic acid C, salvianolic acid B, and neochlorogenic acid) that possess both pharmacological activities and higher exposure levels were regarded as the key effective substances of QSG. Overall, this study provides a comprehensive understanding of the effective components of QSG and could offer a novel strategy for systematically identifying the active components of other TCMs.

## Figures and Tables

**Table 1:** Identification of absorbed components in rat plasma after oral administration of QSG using UHPLC-Q TOF-MS in negative ion mode.

No.	t_R_ (min)	Precursor Ion	Meas. *m*/*z*	Error (ppm)	Molecular Formula	MS2 Fragments	Plausible Identification ^a^	Type ^b^
**1**	0.80	[M−H]^−^	191.0211	7.33	C_6_H_8_O_7_	173.0097, 129.0195, 111.0095	Citric acid	M
**2**	2.05	[M−H]^−^	232.9771	4.21	C_7_H_6_O_7_S	153.0202, 109.0298	Protocatechuic acid-3-*O*-SO_3_	M
**3**	2.07	[M−H]^−^	277.0032	3.14	C_9_H_10_O_8_S	197.0452, 179.0355, 135.0452	Danshensu-3-*O*-SO_3_, Danshensu-4-*O*-SO_3_, or Danshensu-8-*O*-SO_3_	M
**4**	2.21	[M−H]^−^	232.9771	4.21	C_7_H_6_O_7_S	153.0203, 109.0298	Protocatechuic acid-4-*O*-SO_3_	M
**5**	2.68	[M−H]^−^	246.9924	2.55	C_8_H_8_O_7_S	167.0353, 152.0122, 123.0457, 108.0216	Vanillic acid-4-*O*-SO_3_ or isomer	M
**6**	3.02	[M−H]^−^	353.0861	−4.81	C_16_H_18_O_9_	191.0560, 179.0355	Chlorogenic acid ^#^	P
**7**	3.48	[M−H]^−^	246.9923	2.15	C_8_H_8_O_7_S	167.0353, 152.0121, 123.0459, 108.0216	Vanillic acid-4-*O*-SO_3_ or isomer	M
**8**	3.91	[M+HCOO]^−^	209.0466	5.26	C_9_H_8_O_3_	165.0554, 132.0452, 121.0657, 108.0538	Coumaric acid isomer	M
**9**	4.05	[M−H]^−^	375.1303	1.60	C_16_H_24_O_10_	341.9968, 308.1165, 213.0759, 169.0872	10-methoxyl catalpol ^#^	P
**10**	4.31	[M+HCOO]^−^	209.0467	5.74	C_9_H_8_O_3_	165.0558, 121.0657, 108.0537	Coumaric acid	P
**11**	4.39	[M−H]^−^	355.0668	−0.84	C_15_H_16_O_10_	179.0357, 135.0450	Caffeic acid-3-*O*-GluA or Caffeic acid-4-*O*-GluA	M
**12**	4.55	[M+HCOO]^−^	451.1464	1.55	C_17_H_26_O_11_	405.1398	Morroniside ^#^	P
**13**	4.64	[M−H]^−^	353.0884	1.70	C_16_H_18_O_9_	191.0661	Neochlorogenic acid ^#^	P
**14**	4.65	[M−H]^−^	389.1097	2.06	C_16_H_22_O_11_	212.0023	Secologanoside ^#^	P
**15**	4.93	[M−H]^−^	258.9927	3.59	C_9_H_8_O_7_S	179.0359, 135.0450	Caffeic acid-3-*O*-SO_3_ or Caffeic acid-4-*O*-SO_3_	M
**16**	5.10	[M−H]^−^	353.0865	−3.58	C_16_H_18_O_9_	173.0455	Cryptochlorogenic acid ^#^	P
**17**	5.80	[M−H]^−^	369.0830	0.81	C_16_H_18_O_10_	193.0514, 178.0276	(*E*)-Ferulic acid-4-*O*-GluA	M
**18**	5.86	[M−H]^−^	593.1512	0.00	C_27_H_30_O_15_	417.1189, 255.0671, 135.009	Liquiritin-7-*O*-GluA	M
**19**	6.12	[M−H]^−^	369.0829	0.54	C_16_H_18_O_10_	193.0513	(*Z*)-Ferulic acid-4-*O*-GluA	M
**20**	6.24	[M−H]^−^	725.1923	−1.65	C_32_H_38_O_19_	549.1615, 255.067	Liquiritin apioside-7-*O*-GluA	M
**21**	6.34	[M−H]^−^	273.0079	1.76	C_10_H_10_O_7_S	193.0513, 178.0277, 149.0613, 134.0380	(*E*)-Ferulic acid-4-*O*-SO_3_	M
**22**	6.34	[M+HCOO]^−^	403.1254	1.98	C_16_H_22_O_9_	357.1197, 195.0644, 125.0260	Sweroside ^#^	P
**23**	6.42	[M+HCOO]^−^	435.1516	1.84	C_17_H_26_O_10_	389.1442, 227.0933	7-*epi*-loganin ^#^	P
**24**	6.93	[M−H]^−^	621.1467	0.97	C_28_H_30_O_16_	459.0932, 283.0587, 268.0373	Calycosin-7-*O*-D-glucoside-3′-*O*-GluA	M
**25**	7.23	[M−H]^−^	367.1034	−0.27	C_17_H_20_O_9_	191.0565, 173.0457	5-FQA	P
**26**	7.41	[M−H]^−^	403.1256	2.48	C_16_H_22_O_9_	371.0970, 223.0606	Secoxyloganin ^#^	P
**27**	7.59	[M−H]^−^	511.0557	0.98	C_21_H_20_O_13_S	431.0980, 335.0214, 255.0676	Liquiritigenin-7-*O*-GluA-4′-*O*-SO_3_ or Liquiritigenin-7-*O*-SO_3_-4′-*O*-GluA	M
**28**	7.65	[M−H]^−^	273.0079	1.76	C_10_H_10_O_7_S	193.051	(*Z*)-Ferulic acid-4-*O*-SO_3_	M
**29**	8.70	[M−H]^−^	593.1501	−1.85	C_27_H_30_O_15_	417.1184, 255.0677	Isoliquiritin-7-*O*-GluA	M
**30**	8.86	[M+HCOO]^−^	491.1207	−1.15	C_22_H_22_O_10_	283.0612, 135.0460	Calycosin-7-*O*-D-glucoside ^#^	P
**31**	8.86	[M−H]^−^	431.1000	3.71	C_21_H_20_O_10_	255.0671, 135.0084, 119.0502	Liquiritigenin-7-*O*-GluA	M
**32**	9.04	[M−H]^−^	549.1618	0.73	C_26_H_30_O_13_	255.0662, 148, 0144, 135.0085	Liquiritin apioside ^#^	P
**33**	9.10	[M−H]^−^	431.1001	3.94	C_21_H_20_O_10_	255.0673, 135.0091, 119.0504	Liquiritigenin-4′-*O*-GluA	M
**34**	10.25	[M−H]^−^	513.0717	1.75	C_21_H_22_O_13_S	433.1129	Liquiritigenin + H_2_ + GluA + SO_3_	M
**35**	10.61	[M−H]^−^	187.0987	5.88	C_9_H_16_O_4_	169.0885, 143.1077, 125.0978, 102.9495	Azelaic acid	M
**36**	10.70	[M−H]^−^	459.0957	5.23	C_22_H_20_O_11_	283.0609, 268.0378, 239.0355	Calycosin-3′-*O*-GluA or Calycosin-7-*O*-GluA	M
**37**	10.78	[M−H]^−^	187.0986	5.34	C_9_H_16_O_4_	169.0880, 143.1075, 125.0968, 102.9483	Azelaic acid isomer	M
**38**	10.86	[M−H]^−^	417.1213	5.21	C_21_H_22_O_9_	255.0663, 135.0085, 119.0506	Liquiritin ^#^	P, M
**39**	10.94	[M−H]^−^	187.0986	5.34	C_9_H_16_O_4_	169.0886, 125.0967, 102.9483	Azelaic acid isomer	M
**40**	10.95	[M−H]^−^	447.0938	1.12	C_21_H_20_O_11_	285.0395, 151.0035, 133.0300	Luteoloside ^#^	P
**41**	10.96	[M−H]^−^	447.0938	1.12	C_21_H_20_O_11_	271.0617, 151.0046	Naringenin-5-*O*-GluA	M
**42**	11.02	[M−H]^−^	513.0718	1.95	C_21_H_22_O_13_S	433.1128	Isoliquiritigenin + H_2_-7-*O*-GluA-4′-SO_3_	M
**43**	11.26	[M−H]^−^	363.0183	0.88	C_16_H_12_O_8_S	283.0609, 268.0375, 239.0353, 211.0405	Calycosin + SO_3_	M
**44**	11.30	[M−H]^−^	515.1213	3.49	C_25_H_24_O_12_	353.0875, 173.0512, 179.0375	Isochlorogenic acid C ^#^	P
**45**	11.62	[M−H]^−^	549.1252	0.36	C_25_H_26_O_14_	373.0929	Methyl rosmarinic acid-4-*O*-GluA	M
**46**	11.64	[M−H]^−^	513.0718	1.95	C_21_H_22_O_13_S	433.1132	Isoliquiritigenin + H_2_-4′-*O*-GluA-7-SO_3_	M
**47**	11.80	[M−H]^−^	783.2711	−0.77	C_36_H_48_O_19_	607.2211, 461.1642, 193.0512, 175.0396	Angoroside C ^#^	P
**48**	11.98	[M−H]^−^	447.0936	0.67	C_21_H_20_O_11_	271.0617, 243.0662	Naringenin-7-*O*-GluA	M
**49**	12.11	[M+HCOO]^−^	475.1258	2.53	C_22_H_22_O_9_	267.0662, 151.0031	Formononetin-7-*O*-D-glucoside ^#^	P
**50**	12.31	[M−H]^−^	417.1197	1.44	C_21_H_22_O_9_	255.0660, 135.0075, 119.0506	Isoliquiritin ^#^	P, M
**51**	12.33	[M−H]^−^	563.1403	−0.53	C_26_H_28_O_14_	387.1085	3,3′-dimethyl rosmarinic acid-4′-*O*-GluA or 3,3′-dimethyl rosmarinic acid-4-*O*-GluA	M
**52**	12.49	[M−H]^−^	443.0990	1.35	C_22_H_20_O_10_	267.0659, 252.0426	Formononetin-7-*O*-GluA	M
**53**	12.83	[M−H]^−^	717.1450	−1.53	C_36_H_30_O_16_	519.0924, 321.04	Salvianolic acid B ^#^	P
**54**	12.93	[M−H]^−^	433.1140	0.00	C_21_H_22_O_10_	371.1105, 337.0386, 257.0818, 175.0252, 151.0414, 113.0251	Liquiritigenin + H_2_ + GluA	M
**55**	13.31	[M−H]^−^	431.0997	3.02	C_21_H_20_O_11_	255.0668	Isoliquiritigenin-4′-*O*-GluA or Isoliquiritigenin-7-*O*-GluA	M
**56**	13.66	[M−H]^−^	447.0937	0.89	C_21_H_20_O_11_	271.0625	Naringenin-4′-*O*-GluA	M
**57**	13.73	[M−H]^−^	475.0880	−0.42	C_22_H_20_O_12_	433.1134, 421.2078, 267.0668, 252.0428, 223.0399	3′-methoxy-luteolin-7-*O*-GluA or 3′-methoxy-luteolin-4′-*O*-GluA	M
**58**	13.92	[M−H]^−^	475.1258	2.53	C_23_H_24_O_11_	299.0975	C_17_H_16_O_5_ + GluA	M
**59**	14.15	[M−H]^−^	255.0662	−0.39	C_15_H_12_O_4_	135.0085, 119.0499	Liquiritigenin ^#^	P, M
**60**	14.46	[M−H]^−^	475.1254	1.68	C_23_H_24_O_11_	299.0969, 284.0694, 269.0454, 175.0250, 113.0252	C_17_H_16_O_5_ + GluA	M
**61**	15.15	[M−H]^−^	283.0610	−0.71	C_16_H_12_O_5_	135.0075	Calycosin ^#^	P, M
**62**	16.50	[M−H]^−^	271.0610	−0.74	C_15_H_12_O_5_	151.0045	Luteolin ^#^	P, M
**63**	17.82	[M−H]^−^	255.0662	−0.39	C_15_H_12_O_4_	135.0085, 119.0495	Isoliquiritigenin ^#^	P, M
**64**	18.64	[M+HCOO]^−^	829.4589	−0.24	C_41_H_68_O_14_	783.4516	Astragaloside IV ^#^	P
**65**	18.92	[M−H]^−^	267.0662	−0.37	C_16_H_12_O_4_	135.0075	Formononetin ^#^	P, M
**66**	20.16	[M−H]^−^	821.3964	−0.12	C_42_H_62_O_16_	645.3636, 471.2415, 351.0569, 193.0360	Glycyrrhizic Acid ^#^	P
**67**	20.85	[M+HCOO]^−^	871.4682	−1.72	C_43_H_70_O_15_	825.4606	Astragaloside II ^#^	P
**68**	20.97	[M−H]^−^	469.3320	−0.64	C_30_H_46_O_4_	351.0565	Glycyrrhetic Acid ^#^	P
**69**	22.18	[M+HCOO]^−^	871.4697	0.00	C_43_H_70_O_15_	825.4595	Isoastragaloside II	P

(a) Compounds marked with ^#^ were unambiguously identified with standards; GluA, glucuronidation; SO_3_, sulfation. (b) M, metabolite; P, prototype.

**Table 2:** Identification of absorbed components in rat plasma after oral administration of QSG using UHPLC-Q TOF-MS in positive ion mode.

No.	t_R_ (min)	Precursor Ion	Meas. *m*/*z*	Error (ppm)	Molecular Formula	MS2 Fragments	Plausible Identification ^a^	Type ^b^
**70**	2.25	[M+H]^+^	394.2580	−1.52	C_22_H_35_NO_5_	376.2469, 358.2359	Chuanfumine	P
**71**	2.79	[M+H]^+^	424.2691	−0.71	C_23_H_37_NO_6_	406.2570, 388.2478, 374.2310	Senbusine B isomer	M
**72**	3.00	[M+H]^+^	394.2584	−1.01	C_22_H_35_NO_5_	376.2465	Chuanfumine isomer	M
**73**	3.88	[M+H]^+^	486.2681	−3.50	C_24_H_39_NO_9_	468.2590, 454.2426, 436.2304, 422.2171, 404.2058	Mesaconine	P
**74**	3.90	[M+H]^+^	364.2470	−3.29	C_21_H_33_NO_4_	346.2377, 328.2262	16*β*-hydroxycardiopetaline	P
**75**	4.49	[M+H]^+^	408.2738	−1.47	C_23_H_37_NO_5_	390.2614, 358.2389	Isotalatizidine	P
**76**	4.84	[M+H]^+^	500.2845	−1.80	C_25_H_41_NO_9_	468.2583, 450.2482, 436.2389	Aconine	P
**77**	5.01	[M+H]^+^	408.2740	−0.98	C_23_H_37_NO_5_	376.2461, 358.2343	Isotalatizidine isomer	M
**78**	5.11	[M+H]^+^	360.2526	−1.94	C_22_H_33_NO_3_	342.2417, 324.2311	Napelline	P
**79**	5.65	[M+H]^+^	424.2692	−0.47	C_23_H_37_NO_6_	406.2577, 388.2478, 374.2310	Senbusine A isomer	M
**80**	5.70	[M+H]^+^	470.2744	−0.85	C_24_H_39_NO_8_	438.2503, 406.2244	Hypaconine	P
**81**	5.80	[M+H]^+^	454.2783	−3.52	C_24_H_39_NO_7_	436.2703, 404.2486, 386.2265	Fuziline	P
**82**	5.98	[M+H]^+^	408.2743	−0.24	C_23_H_37_NO_5_	376.2475, 358.2372	Isotalatizidine isomer	M
**83**	6.30	[M+H]^+^	438.2847	−0.68	C_24_H_39_NO_6_	420.2732, 402.2577, 388.2467, 370.2377, 356.2208	Neoline	P
**84**	6.76	[M+H]^+^	484.2878	−5.58	C_25_H_41_NO_8_	452.2619	Pseudaconine	P
**85**	7.41	[M+H]^+^	422.2901	0.00	C_24_H_39_NO_5_	390.2628, 372.2449, 358.2387	Talatizamine	P
**86**	8.55	[M+H]^+^	452.2996	−1.55	C_25_H_41_NO_6_	420.2705, 388.2461, 356.2184	Chasmanine	P
**87**	12.01	[M+H]^+^	590.2932	−4.74	C_31_H_43_NO_10_	572.2855, 105.0355	Benzoylmesaconine ^#^	P
**88**	13.12	[M+H]^+^	604.3092	−3.97	C_32_H_45_NO_10_	585.2990, 105.0375	Benzoylaconine ^#^	P
**89**	14.02	[M+H]^+^	574.3018	1.22	C_31_H_43_NO_9_	105.0335	Benzoylhypacoitine ^#^	P
**90**	15.71	[M+H]^+^	616.3116	0.00	C_33_H_45_NO_10_	584.2872, 105.0334	Hypaconitine ^#^	P
**91**	17.68	[M+H]^+^	313.1430	−1.28	C_19_H_20_O_4_	269.1520, 253.0848	Tanshinone IIA + H_2_O	M
**92**	18.23	[M+H]^+^	283.0956	−3.18	C_17_H_14_O_4_	265.0860, 255.0635, 237.0912, 209.0958	Dihydronortanshinone	P
**93**	18.45	[M+H]^+^	297.1488	1.01	C_19_H_20_O_3_	253.1589, 211.1124	Tanshinone IIA + H_2_	M
**94**	18.45	[M+H]^+^	315.1589	−0.63	C_19_H_22_O_4_	297.1494, 253.1595, 237.0934	Tanshinone IIA + H_2_O + H_2_ *	M
**95**	19.70	[M+H]^+^	279.1015	−0.36	C_18_H_14_O_3_	261.0906, 251.1060	Dihydrotanshinone I ^#^	P
**96**	21.10	[M+H]^+^	301.1439	1.66	C_18_H_20_O_4_	283.1325, 265.1208	Tanshinone IIA + H_2_O + H_2_ − CH_3_ *	M
**97**	21.95	[M+H]^+^	295.1320	−3.05	C_19_H_18_O_3_	277.1215	Tanshinone IIA ^#^	P
**98**	22.71	[M+H]^+^	297.1115	−2.02	C_18_H_16_O_4_	279.1008, 261.0904, 233.0964	Tanshinone IIA + O − CH_3_	M
**99**	22.79	[M+H]^+^	327.1229	0.61	C_19_H_18_O_5_	265.1223	Tanshinone IIA + O	M
**100**	23.57	[M+H]^+^	297.1491	2.02	C_19_H_20_O_3_	253.1592, 211.1125	Tanshinone IIA + H_2_	M
**101**	23.57	[M+H]^+^	315.1592	0.32	C_19_H_22_O_4_	297.1497, 279.1393	Tanshinone IIA + H_2_O + H_2_ *	M

(a) Compounds marked with ^#^ were unambiguously identified with standards; compounds marked with * were potential new components. (b) M, metabolite; P, prototype.

**Table 3:** Pharmacokinetic parameters of analytes after oral administration of QSG in rats (*n* = 6).

Compound	T_max_ (h)	C_max_ (ng/mL)	AUC_0→t_ (ng h/mL)	AUC_0→∞_ (ng h/mL)	T_1/2_ (h)	MRT (h)
Chlorogenic acid	0.563 ± 0.125	394.1 ± 6.271	1278 ± 171.4	1418 ± 243.1	9.224 ± 3.268	7.185 ± 0.9780
Neochlorogenic acid	0.398 ± 0.093	68.12 ± 4.044	167.1 ± 68.93	175.7 ± 70.81	4.584 ± 3.300	6.104 ± 2.911
Cryptochlorogenic acid	0.364 ± 0.076	184.1 ± 57.43	532.2 ± 41.45	576.2 ± 58.15	10.84 ± 2.625	8.933 ± 0.5290
Liquiritin apioside	0.432 ± 0.093	50.54 ± 10.68	176.2 ± 22.47	185.7 ± 23.88	7.037 ± 2.092	7.322 ± 0.9520
Luteoloside	0.466 ± 0.076	1.702 ± 0.5580	12.39 ± 2.708	20.86 ± 6.586	21.76 ± 16.85	16.01 ± 1.728
Isochlorogenic acid C	0.334 ± 0.165	33.95 ± 8.980	116.3 ± 39.63	170.5 ± 80.02	16.43 ± 14.14	8.361 ± 4.800
Salvianolic acid B	0.566 ± 0.182	205.3 ± 65.50	1475 ± 187.4	2176 ± 842.3	10.83 ± 5.740	11.08 ± 1.809
Luteolin	0.170 ± 0	12.85 ± 4.120	10.29 ± 0.9300	10.88 ± 0.8670	2.595 ± 1.222	1.839 ± 0.448
Isoliquiritigenin	0.170 ± 0	1.648 ± 0.5850	3.173 ± 0.8430	4.891 ± 2.768	12.37 ± 10.51	7.681 ± 2.173
Formononetin	0.516 ± 0.150	4.310 ± 0.9520	18.51 ± 5.014	27.66 ± 12.24	10.90 ± 9.190	6.795 ± 4.768
Astragaloside IV	0.250 ± 0.0920	12.06 ± 2.010	55.94 ± 26.16	144.9 ± 101.2	14.87 ± 9.060	6.104 ± 3.716

**Table 4:** ECIs for 11 active compounds.

Compound	EC_50_ (μM)	*Wi*	AUC*_i_*	ECI
Cryptochlorogenic acid	1.13	0.3627	576.2	208.98
Chlorogenic acid	8.6	0.0477	1418	67.58
Isochlorogenic acid C	1.09	0.3760	170.5	64.11
Salvianolic acid B	35.15	0.0117	2176	25.37
Neochlorogenic acid	8.81	0.0465	175.7	8.17
Liquiritin apioside	33.16	0.0124	185.7	2.30
Astragaloside IV	76.10	0.0131	144.9	0.78
Luteolin	11.26	0.0364	10.88	0.40
Luteoloside	24.21	0.0169	20.86	0.35
Isoliquiritigenin	10.26	0.0399	4.891	0.20
Formononetin	64.95	0.0063	27.66	0.17

## Data Availability

The original contributions presented in this study are included in the article/Supplementary Material. Further inquiries can be directed to the corresponding authors.

## Supplementary Materials

The following supporting information can be downloaded at https://www.mdpi.com/article/10.3390/ph18101584/s1. Figure S1: MS2 spectrums and proposed fragmentation pathways of liquiritin (A) and isoliquiritin (B); Figure S2: Anti-CHF effects of 10 ingredients on OGD/R-induced H9c2 cells; Table S1: Small molecules for molecular docking; Table S2: Target proteins for molecular docking; Table S3: Molecular docking results of the 49 components with the first 12 target proteins; Table S4: Molecular docking results of the 49 components with the last 12 target proteins; Table S5: Concentrations of 24 analytes in QSG-treated plasma (*n* = 5); Table S6: The effect of compounds on the cytotoxicities of H9c2 cells; Table S7: The retention times (t_R_), fragmentors, and collision energies (CEs) of the analytes; Table S8: Regression equations, linear ranges, and LODs of each analyte; Table S9: Precision and accuracy data for each analyte (*n* = 6); Table S10: Stability data for each analyte (*n* = 6); Table S11: Matrix effect and recovery data for three analytes (*n* = 6); Table S12: Oral relative bioavailabilities of components in QSG.

## Abbreviations

The following abbreviations are used in this manuscript:

AUC	Area under the curve
CE	Collision energy
CHF	Chronic heart failure
DS	Danshen
ECI	Effect–constituent index
EC_50_	Median effective concentration
HSP	Heishunpian
IS	Internal standard
JYH	Jinyinhua
LOD	Limit of detection
MRT	Mean residence time
OGD/R	Oxygen glucose deprivation/re-oxygenation
QC	Quality control
QSG	*Qishen* granule
RSD	Relative standard deviation
S/N	Signal-to-noise ratio
TCMs	Traditional Chinese medicines
XS	Xuanshen
ZGC	Zhigancao
ZHQ	Zhihuangqi
