# Supplementary material for: An Efficacy- and In Vivo Exposure-Oriented Integrated Study to Investigate the Effective Components of Qishen Granule
Source: Pharmaceuticals (Basel). 2025 Oct 20;18(10):1584. doi: 10.3390/ph18101584 (PMC12567096; doi:10.3390/ph18101584)
Supplement: Supplementary file 1 [file pharmaceuticals-18-01584-s001.zip › pharmaceuticals-3864700-supplementary.pdf]

Supplemental information

An efficacy- and *in vivo* exposure-oriented integrated study to investigate the effective components of *Qishen* Granule

Yueting Li [1,2], Tengteng Wang [1,2], Chao Cheng [1,2], Yingying Huo [1,2], Ying Tan [1,2], Yifan Xu [1,2],Jiale Gao [2,3], Jie Liu [2,3*], Hongbin Xiao [2,3*]

[1] School of Chinese Materia Medica, Beijing University of Chinese Medicine, Beijing 100029, China; [2] Research Center for Chinese Medicine Analysis and Transformation, Beijing University of Chinese Medicine, Beijing 100029, China; [3] Beijing Research Institute of Chinese Medicine, Beijing University of Chinese Medicine, Beijing 100029, China.

The list of contents

No.	Content
**Figure S1**	MS2 spectrums and proposed fragmentation pathways of liquiritin (A) and isoliquiritin (B).
**Figure S2**	Anti-CHF effects of 10 ingredients on OGD/R induced H9c2 cells.
**Table S1**	Small molecules for molecular docking.
**Table S2**	Target proteins for molecular docking.
**Table S3**	Molecular docking results of the 49 components with the first 12 target proteins.
**Table S4**	Molecular docking results of the 49 components with the last 12 target proteins.
**Table S5**	Concentrations of 24 analytes in QSG treated plasma (*n* = 5).
**Table S6**	The effect of compounds on the cytotoxicities of H9c2 cells.
**Table S7**	The retention times (t_R), fragmentors, collision energies (CEs) of the analytes.
**Table S8**	Regression equations, linear ranges and LODs of each analyte.
**Table S9**	Precision and accuracy data for each analyte (*n* = 6).
**Table S10**	Stability data for each analyte (*n* = 6).
**Table S11**	Matrix effect and recovery data for three analytes (*n* = 6).
**Table S12**	Oral relative bioavailabilities of components in QSG.

A

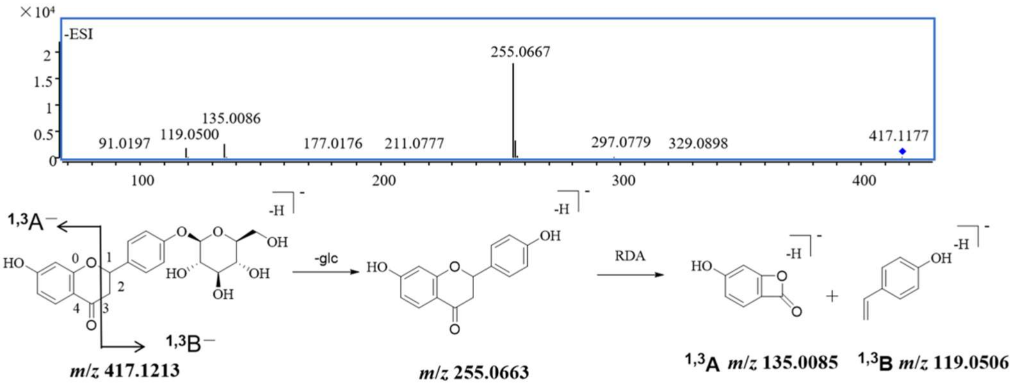

B

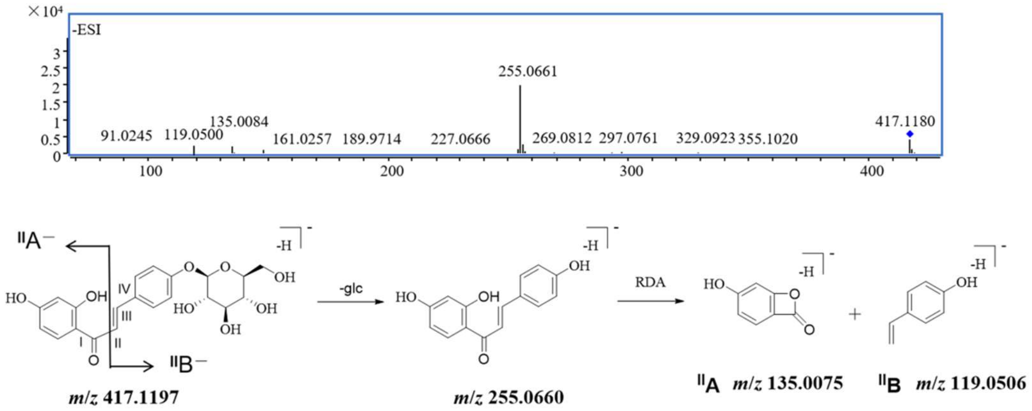

Figure S1 MS2 spectrums and proposed fragmentation pathways of liquiritin (A) and isoliquiritin (B).

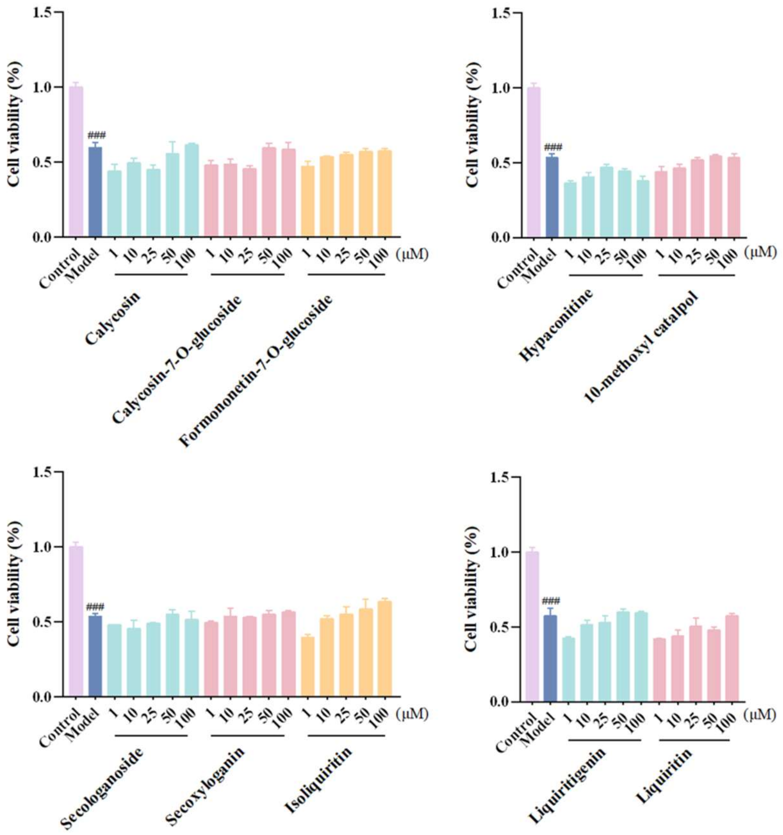

Figure S2 Anti-CHF effects of 10 ingredients on OGD/R induced H9c2 cells.

Data were expressed as the mean ± SD (n = 6; ###, $p < 0.001$, compared with the control group).

Table S1 Small molecules for molecular docking

NO[a]	Small molecules	NO[a]	Small molecules	NO[a]	Small molecules
**1**	Citric acid isomer	**28**	(*Z*)-Ferulic acid-4-*O*-SO_3	**62**	Luteolin
**2**	Protocatechuic acid-3-*O*-SO_3	**29**	Isoliquiritin-7-*O*-GluA	**63**	Isoliquiritigenin
**3-1** [b]	Danshensu-3-*O*-SO_3	**30**	Calycosin-7-*O*-glucoside	**64**	Astragaloside IV
**3-2** [b]	Danshensu-4-*O*-SO_3	**31**	Liquiritigenin-7-*O*-GluA	**65**	Formononetin
**3-3** [b]	Danshensu-8-*O*-SO_3	**32**	Liquiritin apioside	**66**	Glycyrrhizic Acid
**4**	Protocatechuic acid-4-*O*-SO_3	**33**	Liquiritigenin-4'-*O*-GluA	**67**	Astragaloside II
**5**	Vanillic acid-4-*O*-SO_3	**35**	Azelaic acid	**68**	Glycyrrhetic acid
**6**	Chlorogenic acid	**36-1** [b]	Calycosin-3′-*O*-GluA	**69**	Isoastragaloside II
**9**	10-methoxyl catalpol	**36-2** [b]	Calycosin-7-*O*-GluA	**73**	Mesaconine
**10**	Coumaric acid	**38**	Liquiritin	**74**	16*β*-hydroxycardiopetaline
**11-1** [b]	Caffeic acid-3-*O*-GluA	**40**	Luteoloside	**75**	Isotalatizidine
**11-2** [b]	Caffeic acid-4-*O*-GluA	**41**	Naringenin-4′-*O*-GluA	**76**	Aconine
**12**	Morroniside	**43-1** [b]	Calycosin-7-*O*-SO_3	**78**	Napelline
**13**	Neochlorogenic acid	**43-2** [b]	Calycosin-3′-*O*-SO_3	**80**	Hypaconine
**14**	Secologanoside	**44**	Isochlorogenic acid C	**81**	Fuziline
**15-1** [b]	Caffeic acid-3-*O*-SO_3	**45**	Methyl rosmarinic acid-4-*O*-GluA	**83**	Neoline
**15-2** [b]	Caffeic acid-4-*O*-SO_3	**47**	Angoroside C	**84**	Pseudaconine
**16**	Cryptochlorogenic acid	**48**	Naringenin-5-*O*-GluA	**85**	Talatizamine
**17**	(*E*)-Ferulic acid-4-*O*-GluA	**49**	Formononetin-7-*O*-glucoside	**86**	Chasmanine
**18**	Liquiritin-7-*O*-GluA	**50**	Isoliquiritin	**87**	Benzoylmesaconine
**19**	(*Z*)-Ferulic acid-4-*O*-GluA	**51-1** [b]	3,3′-dimethyl rosmarinic acid-4′-*O*-GluA	**88**	Benzoylaconine

NO[a]	Small molecules	NO[a]	Small molecules	NO[a]	Small molecules
**20**	Liquiritin apioside-7-*O*-GluA	**51-2** [b]	3,3′-dimethyl rosmarinic acid-4-*O*-GluA	**89**	Benzoylhypacoitine
**21**	(*E*)-Ferulic acid-4-*O*-SO_3	**52**	Formononetin-7-*O*-GluA	**90**	Hypaconitine
**22**	Sweroside	**55-1** [b]	Isoliquiritigenin-4′-*O*-GluA	**92**	Dihydronortanshinone
**23**	7-*epi*-loganin	**55-2** [b]	Isoliquiritigenin-7-*O*-GluA	**93**	15,16-hydrotanshinone IIA
**24**	Calycosin-7-*O*-D-glucoside-3′-*O*-GluA	**56**	Naringenin-7-*O*-GluA	**94**	Tanshinone IIA + H_2O + H_2
**26-1** [b]	Secoxyloganin	**53**	Salvianolic acid B	**95**	Dihydrotanshinone I
**26-2** [b]	Secologanoside-7-methyl ester	**57-1** [b]	3'-methoxy-luteolin-7-*O*-GluA	**97**	Tanshinone IIA
**26-3** [b]	Kingiside	**57-2** [b]	3'-methoxy-luteolin-4′-*O*-GluA	**99**	Tanshinone IIA+O
**27-1** [b]	Liquiritigenin-7-*O*-GluA-4′-*O*-SO_3	**59**	Liquiritigenin		
**27-2** [b]	Liquiritigenin-7-*O*-SO_3-4′-*O*-GluA	**61**	Calycosin		

a) The notation for compounds refers to Tables 1&2. b) All possible substitution structures are listed for compound that could not determine the substitution positions

Table S2 Target proteins for molecular docking

Protein	Full name	PDB code
ACE	Angiotensin-Converting Enzyme	6H5W
ADORA1	Adenosine A1 Receptor	6D9H
ADORA2A	Adenosine A2a Receptor	5IU4
ADRB2	Adrenergic Receptor Beta 2	2RH1
AGTR1	Angiotensin II Receptor Type 1	6OS2
AGT	Angiotensinogen	5M3Y
AR	Androgen Receptor	2PIT
CACNA1G	Calcium Voltage-Gated Channel Subunit Alpha1 G	6KZP
CASP3	Caspase 3	1NME
CES1	Carboxylesterase 1	2H7C
CHRM2	Cholinergic Receptor Muscarinic 2	5ZK8
COX2	Cytochrome C Oxidase Assembly Factor COX2	5F1A
CXCR4	C-X-C Motif Chemokine Receptor 4	3ODU
CYBB	Cytochrome B-245 Beta Chain	3A1F
ESR1	Estrogen Receptor 1	6SBO
ESR2	Estrogen Receptor 2	1QKM
F10	Coagulation Factor X	2Y5F
F2	Coagulation Factor II	1LHC
HTR2B	5-Hydroxytryptamine Receptor 2B	4IB4
MMP-9	Matrix Metallopeptidase 9	6ESM
MTTP	Microsomal Triglyceride Transfer Protein	6I7S
NOS3	Nitric Oxide Synthase 3	1M9J
P53	Tumor Protein P53	5A7B
PDE1B	Phosphodiesterase 1B	5W6E
PLA2	Phospholipase A2	6Q42
PPARA	Peroxisome Proliferator Activated Receptor Alpha	3VI8
PTGS1	Prostaglandin-Endoperoxide Synthase 1	6Y3C
REN	Angiotensinogenase Renin	2V0Z
RhoA	Ras Homolog Family Member A	1A2B
ROCK1	Rho Associated Coiled-Coil Containing Protein Kinase 1	3V8S
ROCK2	Rho Associated Coiled-Coil Containing Protein Kinase 2	4L6Q
TGF-B1	Transforming Growth Factor Beta 1	5VQP
THRA	Thyroid Hormone Receptor Alpha	2H79
TNF	Tumor Necrosis Factor	2AZ5
V1AR	Vasopressin V1a Receptor	1YTV
XDH	Xanthine Dehydrogenase	2E1Q

Table S3 Molecular docking results of the 49 components with the first 12 target proteins

NO[a]	ACE	ADORA1	ADORA2A	ADRB2	CASP3	CES1	CHRM2	CXCR4	ESR1
**2**	–	–	–	–	–	–	–	–	–
**3-1**	–	–	7.278	–	–	7.1933	–	–	–
**3-2**	–	–	8.0278	–	–	7.6521	–	7.9267	–
**3-3**	–	–	–	–	–	7.4621	–	–	–
**4**	–	–	–	–	–	–	–	–	–
**5**	–	–	–	–	–	–	–	–	–
**6**	–	–	–	–	–	–	–	–	–
**9**	–	7.3665	7.4484	–	–	7.6878	–	–	–
**11-1**	7.5441	7.0721	7.2503	–	–	–	10.0798	–	7.3598
**11-2**	–	–	–	–	–	–	–	–	7.1655
**12**	–	–	–	–	–	7.9136	–	–	–
**13**	–	–	–	–	–	–	–	–	–
**14**	7.4025	7.3848	–	–	–	–	7.3053	–	7.3745
**15-1**	–	–	–	–	–	–	–	–	–
**15-2**	–	–	–	–	–	–	–	–	–
**16**	8.4609	–	–	–	–	–	–	–	–
**17**	–	7.7568	7.5888	7.6203	–	–	–	–	–
**18**	8.7462	–	–	–	–	–	–	–	–
**19**	7.7655	10.3989	–	7.2806	–	–	–	7.1497	–
**20**	7.1723	–	–	8.3024	–		–	8.3437	–
**23**	–	–	–	7.0375	–	–	–	–	–
**24**	7.1854	–	–	–	–	–	–	–	–
**26-1**	8.3233	–	–	7.0377	–	8.062	–	–	–
**26-2**	7.0058	–	–	–	–	–	7.0048	–	–
**26-3**	8.7415	–	–	–	–	–	–	–	–
**27-1**	7.6214	–	–	–	–	–	–	7.1236	–

NO[a]	ACE	ADORA1	ADORA2A	ADRB2	CASP3	CES1	CHRM2	CXCR4	ESR1	F10	F2	HTR2B
**27-2**	7.2377	–	8.8219	–	–	–	–	–	–	–	–	–
**28**	–	7.2221	7.2045	8.0359	–	–	–	–	–	–	–	8.5234
**29**	9.3889	–	–	–	–	–	–	8.0727	7.6317	–	–	7.5122
**30**	–	–	–	7.1534	–	–	–	–	–	–	–	–
**31-1**	–	–	–	–	–	7.112	–	–	–	7.5486	7.3214	–
**31-2**	–	–	–	–	–	8.3914	–	–	–	–	–	–
**32**	–	–	–	–	–	8.0435	–	9.213	–	8.3405	–	8.3018
**36-1**	–	–	–	9.7863	–	7.8409	–	7.1876	–	–	7.9239	–
**36-2**	–	–	–	7.4632	–	–	–	–	–	–	–	7.6164
**38**	8.0084	–	–	–	–	–	7.5105	–	–	–	–	–
**40**	7.1717	7.0335	–	–	–	–	–	7.0314	–	–	8.0845	8.3598
**41**	–	–	–	–	–	–	7.1627	–	–	–	–	–
**43-1**	–	–	7.3589	–	–	–	–	–	–	–	–	–
**43-2**	–	–	8.7866	–	–	–	–	–	–	–	7.1375	–
**44**	–	–	–	–	–	7.2433	–	–	–	–	–	7.9596
**45**	9.1456	8.7939	8.962	8.5203	–	–	–	7.6415	9.3117	7.4272	7.6844	8.8328
**47**	9.1836	–	–	–	–	7.1303	–	10.8701	–	11.5226	7.6948	7.2991
**48**	7.0701	–	–	7.6942	–	–	–	–	–	–	7.2535	–
**49**	–	–	–	7.0821	–	–	–	8.1527	–	–	7.3949	–
**50**	7.0961	–	–	7.716	–	–	–	7.8788	–	–	–	–
**51-1**	9.2681	11.3255	7.5078	7.6067	6.9785	7.9630	–	8.3276	7.9872	7.9799	–	8.6740
**51-2**	8.3386	10.145	9.2035	9.0039	7.2994	7.3126	–	8.4888	8.3058	8.0277	9.6997	10.3151
**52**	7.8067	–	8.1143	–	–	–	–	–	–	–	–	–
**53**	8.7934	–	–	8.8372	8.2696	7.9857	–	11.5247	–	7.6371	8.6106	–
**55-1**	7.7657	–	7.5787	–	7.474	–	–	8.9952	7.9121	–	–	8.0717
**55-2**	–	–	–	7.6527	–	–	–	7.3501	8.9278	–	–	7.3057
**56**	–	–	7.3262	–	–	–	–	–	–	–	–	8.2645

NO[a]	ACE	ADORA1	ADORA2A	ADRB2	CASP3	CES1	CHRM2	CXCR4	ESR1	F10	F2	HTR2B
**57-1**	10.0011	–	8.0194	–	–	–	–	7.5384	–	7.1296	7.4757	7.2901
**57-2**	–	–	–	–	–	8.0347	–	–	–	–	9.8565	–
**59**	–	–	–	–	–	7.2495	–	–	–	–	–	–
**61**	–	–	7.2381	–	–	–	–	–	–	–	–	–
**62**	–	–	–	–	–	–	–	–	–	–	–	–
**63**	–	–	–	–	–	–	–	–	–	–	–	–
**64**	–	–	–	–	–	–	–	–	–	–	–	–
**65**	–	–	–	–	–	–	–	–	–	7.3178	–	–
**90**	9.4236	–	–	–	–	–	–	–	–	–	–	–

a) The notation for compounds refers to Tables 1&2. "–" ：Total Score < 7

Table S4 Molecular docking results of the 49 components with the last 12 target proteins

NO[a]	MMP-9	MTTP	NOS3	PDE1B	PPARA	REN	RhoA	ROCK1	ROCK2	TGF-B1	V1AR	XDH
**2**	–	–	–	–	–	–	7.3134	–	–	–	–	–
**3-1**	–	–	–	–	–	–	–	–	–	–	7.7575	7.2660
**3-2**	–	–	–	–	–	–	8.8296	–	–	–	7.9306	–
**3-3**	–	–	–	–	–	–	7.7649	–	–	8.5434	9.2076	7.0017
**4**	–	–	–	–	–	–	–	–	–	–	–	7.0737
**5**	–	–	–	–	–	–	–	–	–	–	–	–
**6**	7.3908	–	–	–	–	–	–	–	–	–	–	7.1158
**9**	–	–	–	–	–	–	–	–	–	–	8.1820	–
**11-1**	7.6056	–	–	–	–	–	7.4061	7.9174	–	–	8.1774	–
**11-2**	–	–	–	7.5343	–	–	7.0972	–	–	–	9.0870	–
**12**	–	–	–	–	–	–	–	–	–	–	12.8204	–
**13**	8.3159	–	–	–	–	–	–	–	–	–	7.1681	–
**14**	–	–	–	–	–	–	–	–	7.0401	–	9.4634	–
**15-1**	–	–	–	–	–	–	7.0224	–	–	–	8.1194	7.2918
**15-2**	–	–	–	–	–	–	–	–	–	–	–	7.1084
**16**	–	–	–	–	–	–	–	–	–	–	8.3590	–
**17**	–	–	–	7.1374	7.0671	–	8.6031	–	–	–	7.7336	7.3880
**18**	–	–	8.4590	–	8.7742	–	–	10.0943	7.0896	–	–	–
**19**	7.3762	–	–	–	–	–	7.7564	7.7766	–	–	7.3547	–
**20**	–	9.5424	8.6459	–	–	–	–	9.1551	–	–	–	–
**23**	–	–	–	–	–	–	–	–	–	–	8.2046	–
**24**	–	–	–	7.1632	–	–	–	–	–	–	8.8970	–
**26-1**	–	–	–	–	–	7.5065	–	–	–	–	9.3870	–

NO[a]	MMP-9	MTTP	NOS3	PDE1B	PPARA	REN	RhoA	ROCK1	ROCK2	TGF-B1	V1AR	XDH
**26-2**	–	–	–	–	–	–	7.8936	–	–	–	9.5613	–
**26-3**	–	–	–	–	–	–	–	–	–	–	10.6144	–
**27-1**	–	–	–	–	–	7.5575	7.6218	8.9259	–	–	9.7548	–
**27-2**	–	–	7.3377	–	–	–	–	–	–	–	10.9034	–
**28**	–	–	–	–	–	–	8.4200	7.5138	–	–	8.2426	–
**29**	–	–	9.0504	–	10.1529	9.4265	8.1345	8.0626	–	–	9.2683	–
**30**	8.5804	7.3864	7.1826	7.2028	–	–	–	–	–	–	–	–
**31-1**	–	–	8.0688	–	–	–	7.2220	–	–	–	7.5892	–
**31-2**	–	–	–	–	–	–	–	–	–	–	7.9332	–
**32**	–	–	–	–	–	7.0982	–	–	–	–	13.4843	–
**36-1**	–	–	–	–	–	–	8.6765	–	–	–	8.4084	8.9717
**36-2**	8.1118	–	–	8.2314	–	–	8.9937	–	–	–	8.0852	–
**38**	–	–	–	–	–	–	–	–	–	–	7.5456	–
**40**	–	–	–	–	–	–	8.5798	10.1661	7.0598	7.0111	10.5705	7.3963
**41**	–	–	–	–	–	–	–	–	–	–	7.2357	–
**43-1**	9.8161	–	–	8.9967	–	–	9.3090	–	–	–	–	–
**43-2**	–	–	–	–	–	–	7.0031	–	–	–	7.4006	7.1635
**44**	–	–	9.0955	7.8106	–	9.0770	8.8819	10.6659	8.4536	–	–	–
**45**	7.3516	8.7678	8.7457	7.4926	9.7345	8.7234	–	–	8.3878	–	–	–
**47**	9.1199	8.2099	12.8979	11.3208	8.9825	10.8393	7.3914	10.2588	11.2636	–	15.6013	–
**48**	–	–	–	7.1638	–	–	7.6035	–	8.1947	–	–	–
**49**	7.5047	–	–	8.4413	–	–	–	–	–	–	8.5403	–
**50**	7.0799	–	–	–	7.9152	–	–	–	–	–	9.8689	9.3401
**51-1**	–	–	–	–	–	–	7.3892	7.4624	8.2368	–	10.1990	8.6240
**51-2**	7.8448	–	7.9889	7.2163	7.1664	7.6762	9.7822	9.6439	7.2231	8.2506	9.1466	10.6302

NO[a]	MMP-9	MTTP	NOS3	PDE1B	PPARA	REN	RhoA	ROCK1	ROCK2	TGF-B1	V1AR	XDH
**52**	–	–	7.3660	–	7.2193	–	7.3593	7.6236	7.1687	–	8.6657	–
**53**	10.8710	7.8279	9.2773	8.7553	11.2019	11.9067	7.6935	10.3145	10.2734	–	12.5368	10.9803
**55-1**	8.9986	–	–	–	7.0140	–	8.6191	7.5145	7.1758	–	8.6738	–
**55-2**	–	–	–	–	7.1811	7.2216	–	–	–	–	10.6626	–
**56**	–	–	–	–	7.2032	7.0195	7.1526	7.5529	–	–	7.1369	–
**57-1**	–	–	–	–	7.4278	–	9.8266	–	–	–	–	–
**57-2**	–	8.1848	–	8.2804	7.9498	–	9.4793	7.6824	7.5441	–	8.6434	–
**59**	–	–	–	–	–	–	–	–	–	–	–	–
**61**	–	–	–	–	–	–	–	–	–	7.6436	–	–
**62**	–	–	–	–	–	–	–	–	–	–	–	8.7040
**63**	–	–	–	–	–	–	–	–	–	7.7876	–	–
**64**	–	–	7.9153	–	–	–	–	–	7.4670	–	7.1223	–
**65**	–	–	–	–	–	–	–	–	–	–	–	7.6856
**90**	–	–	–	–	–	–	–	–	–	–	–	–

a) The notation for compounds refers to Table 1&2. "–" ：Total Score < 7

Table S5 Concentrations of 24 analytes in QSG treated plasma (n = 5)

Analyte	Concentration (ng/mL)								
	**0.08 h**	**0.17 h**	**0.33 h**	**0.5 h**	**1 h**	**2 h**	**4 h**	**8 h**	**12 h**
**Calycosin**	0.966±0.737	2.155±0.363	3.224±1.497	2.512±0.672	1.704±0.563	0.825±0.375	0.589±0.460	0.737±0.203	0.647±0.195
**Calycosin-7-*O*-glucoside**	1.161±0.988	2.081±1.576	9.994±1.861	8.612±0.681	3.192±1.624	3.096±2.601	1.198±1.103	1.241±0.963	0.536±0.271
**Formononetin**	0.740±0.932	2.809±1.237	3.781±0.871	4.234±0.976	2.771±1.064	1.453±0.436	1.028±0.395	0.656±0.349	0.289±0.213
**Formononetin-7-*O*-glucoside**	2.551±2.137	5.216±2.303	11.525±4.246	9.937±3.169	6.198±2.872	2.708±0.988	1.088±0.951	0.853±0.489	0.171±0.342
**Astragaloside IV**	-	7.10±5.941	6.739±5.744	8.655±2.757	3.017±3.507	3.783±2.015	0.710±1.991	1.337±1.728	2.200±2.015
**Salvianolic B**	68.337±49.223	115.490±60.235	128.948±38.720	174.763±84.875	83.989±21.934	47.989±9.996	34.225±16.349	91.217±8.136	56.609±10.151
**Hypaconitine**	0.048±0.019	0.202±0.096	0.228±0.021	0.308±0.041	0.340±0.066	0.331±0.081	0.212±0.084	0.071±0.049	0.005±0.013
**Luteolin**	5.839±1.287	13.610±4.120	9.211±2.098	4.371±1.587	2.410±0.697	0.911±0.249	0.251±0.183	0.188±0.101	0.042±0.044
**Luteoloside**	0.343±0.313	0.335±0.070	1.118±0.238	1.384±0.727	0.439±0.328	0.266±0.168	0.093±0.163	0.515±0.139	0.234±0.265
**Neochlorogenic acid**	6.765±1.942	14.616±13.731	66.644±5.290	60.064±10.063	24.771±9.507	9.851±7.030	6.874±2.587	10.585±4.645	7.356±4.074
**Cryptochlorogenic acid**	17.855±11.504	95.627±23.461	159.318±60.161	135.955±20.918	54.572±22.599	29.505±18.460	18.424±6.281	21.857±6.099	12.189±3.932
**Chlorogenic acid**	66.231± 20.073	99.061±0.162	363.231±37.857	394.981±7.098	170.435±35.745	77.157±7.263	68.117±20.995	51.389±5.490	39.119±14.876
**Isochlorogenic acid C**	12.829±7.386	23.904±9.523	24.375±12.494	18.491±8.090	11.005±7.334	7.777±3.293	2.637±2.154	7.703±2.363	1.848±1.625
**7-*epi*-loganin**	-	-	0.650±0.301	1.730±0.490	1.500±0.298	-	-	-	-
**Secoxyloganin**	-	-	1.247±0.459	3.173±1.274	2.317±1.028	-	-	-	-
**Secologanoside**	-	-	2.382±1.203	4.124±1.235	2.135±1.024	1.274±0.246	-	-	-
**10-methoxyl**	-	-	3.215±1.293	2.315±0.283	1.293±0.135	-	-	-	-

**catalpol**									
**Morroniside**	-	0.702 0.290	1.042±0.059	3.052±0.802	2.401±0.829	-	-	-	
**Angoroside C**	-	-	1.430±0.689	4.290±1.023	3.109±0.501	1.029±0.203	-	-	
**Isoliquiritigenin**	0.690±0.227	1.648±0.585	0.933±0.388	0.454±0.151	0.355±0.170	0.179±0.041	0.231±0.054	0.053 ±0.038	0.124±0.092
**Liquiritigenin**	1.163±0.835	3.082±0.977	4.442±0.809	4.378±0.959	3.255±1.246	2.336±1.150	2.064±0.888	2.239±1.209	2.987±1.135
**Isoliquiritin**	0.332±0.496	1.244±0.874	2.307±0.456	2.538±0.684	1.307±0.788	0.456±0.573	0.075±0.309	0.078±0.159	0.158±0.323
**Liquiritin**	2.068±1.321	11.787±10.878	24.380±5.573	26.784±4.419	17.820±5.293	8.298±2.485	4.024±3.297	2.173±0.609	1.363±2.740
**Liquiritin apioside**	4.615±2.872	18.791±17.432	42.303±13.041	43.543±9.903	20.141±7.771	13.225±5.233	8.826±3.566	7.075±1.340	4.785±0.620

"–" ：Concentrations were below the lower limit of quantification

Table S6 The effect of compounds on the cytotoxicities of H9c2 cells (n=3)

Compound	Inhibition rate（%）[a]		
	**25 μM**	**50 μM**	**100 μM**
Formononetin-7-*O*-glucoside	0.8 ± 0.9	9.1 ± 4.3	11.1 ± 2.3
Formononetin	10.7 ± 4.5	10.8 ± 5.2	13.5 ± 2.2
Calycosin-7-*O*-glucoside	-1.2 ± 3.5	7.8 ± 6.4	11.8 ± 6.1
Calycosin	6.8 ± 3.9	6.3 ± 8.3	9.3 ± 4.2
Astragaloside IV	-6.7 ± 7.1	7.0 ± 2.1	15.0 ± 2.3
Salvianolic acid B	-38.9 ± 5.2	-17.1 ± 7.2	0.1 ± 3.2
Hypaconitine	13.7 ± 4.1	20.8 ± 3.2	32.8 ± 1.2
Luteoloside	-4.2 ± 4.7	8.1 ± 5.7	4.1 ± 1.7
Luteolin	12.2 ± 4.0	13.1 ± 7.2	18.1 ± 4.2
Chlorogenic acid	-1.6 ± 4.9	12.0 ± 1.7	15.0 ± 4.1
Neochlorogenic acid	-12.8 ± 1.3	3.8 ± 2.2	5.7 ± 2.3
Cryptochlorogenic acid	-16.2 ± 4.2	-3.4 ± 0.2	5.2 ± 0.4
Isochlorogenic acid C	-11.8 ± 6.3	-16.6 ± 1.3	-6.3 ± 2.3
7-*epi*-loganin	2.8 ± 0.9	7.1 ± 4.3	10.3 ± 3.5
Secoxyloganin	3.5 ± 1.2	7.9 ± 3.3	9.9 ± 2.7
Secologanoside	-15.6 ± 7.9	11.0 ± 7.7	19.1 ± 3.7
10-methoxyl catalpol	2.8 ± 0.9	5.1 ± 4.3	9.1 ± 4.2
Morroniside	-10.6 ± 7.6	2.1 ± 5.6	8.1 ± 3.9
Angoroside C	3.6 ± 4.5	12.7 ± 5.1	18.7 ± 1.1
Liquiritin	-7.4 ± 7.5	11.6 ± 0.6	17.6 ± 1.2
Isoliquiritin	-18.3 ± 3.6	8.5 ± 2.9	13.5 ± 2.3
Isoliquiritigenin	-14.9 ± 9.3	-1.2 ± 7.6	7.2 ± 4.2
Liquiritin apioside	-25.2 ± 3.5	-16.2 ± 3.6	-6.1 ± 2.5
Liquiritigenin	-4.4 ± 2.2	2.3 ± 3.3	8.5 ± 3.6

Table S7 The retention times (t_R), fragmentors, collision energies (CEs) of the analytes

NO.	Analyte[a]	t_R (min)	Ion transition Precursor>product ion[a]	Fragmentor (V)	CE (eV)	Ion mode
**6**	Chlorogenic acid	4.42	**353.1 > 191.3**; 353.1 > 179.3	75	15	Negative
**13**	Neochlorogenic acid	6.24	**353.1 > 191.3**; 353.1 > 179.3	75	20	Negative
**16**	Cryptochlorogenic acid	6.66	**353.1 > 173.3**; 353.1 > 179.3	85	15	Negative
**32**	Liquiritin apioside	10.11	**549.1 > 255.4**; 549.1 > 135.1	210	35	Negative
**40**	Luteoloside	11.13	**447.0 > 285.4**; 447.0 > 151.0	175	30	Negative
**44**	Isochlorogenic acid C	12.27	**515.0 > 179.3**; 515.0 > 173.3	155	35	Negative
**53**	Salvianolic acid B	12.88	**717.1 > 519.2**; 717.1 >321.0	145	15	Negative
**62**	Luteolin	15.01	**285.0 > 133.3**; 285.0 > 175.2	165	40	Negative
**63**	Isoliquiritigenin	16.60	**255.1 > 119.3**; 255.1 >135.3	150	15	Negative
**65**	Formononetin	17.42	**267.0 > 252.4**; 267.0 > 223.4	135	20	Negative
**IS**	Icariin	17.65	**675.3 > 513.4**; 675.3 > 367.5	205	10	Negative
**64**	Astragaloside IV	21.69	**783.3 > 489.7**; 783.3 > 621.4	195	25	Negative

[a]: two ion pairs were optimized for each analyte, and the ion transitions in bold were implemented for quantitative analysis.

Table S8 Regression equations, linear ranges and LODs of each analyte

Analyte	Regression equation	R^2	Linear range (ng/mL)	LOD（ng/mL）
Chlorogenic acid	y = 16.2 x + 0.103	0.9994	0.320 – 200	0.10
Neochlorogenic acid	y = 6.82 x + 0.388	0.9991	0.640 – 400	0.20
Cryptochlorogenic acid	y = 5.85 x + 0.0756	0.9989	0.640 – 400	0.40
Liquiritin apioside	y = 2.23 x + 0.00836	0.9997	0.320 – 400	0.10
Luteoloside	y = 5.08 x + 0.00765	0.9991	0.128 – 200	0.020
Isochlorogenic acid C	y = 0.640 x + 0.0130	0.9995	1.60 – 400	0.40
Salvianolic acid B	y = 1.39 x + 0.00380	0.9998	3.20 – 2000	0.20
Luteolin	y = 0.274 x + 0.00973	0.9988	0.064– 80	0.010
Isoliquiritigenin	y = 0.435 x + 0.045	0.9989	0.0256 – 40	0.010
Formononetin	y = 9.66 x + 0.0325	0.9998	0.320 – 200	0.10
Astragaloside IV	y = 5.71 x – 0.000573	0.9973	3.20 – 400	1.0

Table S9 **Precision and accuracy data for each analyte (*n* = 6)**

Analyte	Spiked (ng/mL)	Intra-day		Inter-day	
		Accuracy（%）	RSD（%）	Accuracy（%）	RSD（%）
Chlorogenic acid	0.64	112.78	9.63	103.83	12.02
	16	85.10	2.41	89.04	7.76
	80	85.34	1.23	87.72	2.90
Neochlorogenic acid	3.2	87.21	5.83	87.08	6.40
	16	89.12	1.49	92.33	4.69
	80	87.61	1.10	100.66	2.77
Cryptochlorogenic acid	3.2	114.11	2.13	105.45	10.99
	16	100.81	3.18	93.89	9.98
	80	95.62	2.58	90.76	7.39
Liquiritin apioside	0.64	113.84	5.10	114.74	2.70
	16	114.04	2.91	111.53	5.05
	80	114.70	2.90	112.56	4.03
Luteoloside	0.64	110.37	4.70	107.60	3.86
	16	110.38	3.08	108.28	3.37
	80	106.49	2.64	106.06	3.41
Isochlorogenic acid C	3.2	97.18	9.04	92.14	9.20
	16	90.13	6.99	102.00	5.07
	80	101.24	2.54	108.70	4.74
Salvianolic acid B	16	108.67	1.02	106.51	5.88
	80	85.16	4.63	85.44	5.40
	400	87.21	2.80	98.21	5.70
Luteolin	0.32	103.97	5.91	104.09	5.58

**Analyte**	**Spiked (ng/mL)**	**Intra-day**		**Inter-day**	
		**Accuracy（%）**	**RSD（%）**	**Accuracy（%）**	**RSD（%）**
	1.6	109.23	2.22	105.49	4.08
	16	109.41	2.68	107.97	3.14
	0.064	85.19	3.89	85.57	7.42
Isoliquiritigenin	1.6	97.35	2.70	90.91	7.42
	16	98.96	2.01	93.32	5.91
	0.64	105.49	6.28	112.58	8.68
Formononetin	16	91.54	2.54	94.07	2.59
	80	91.86	2.48	93.35	3.12
	16	88.82	7.85	85.32	4.66
Astragaloside IV	40	106.00	1.87	107.50	3.20
	80	114.19	13.68	111.99	10.20

Table S10 Stability data for each analyte (*n* = 6)

Analyte	Spiked (ng/mL)	Short-term		Long-term		Three-freeze-thraw	
		Accuracy（%）	RSD（%）	Accuracy（%）	RSD（%）	Accuracy（%）	RSD（%）
Chlorogenic acid	0.64	91.18	8.43	87.95	1.73	93.23	6.07
	16	91.15	4.37	85.66	6.88	91.86	4.52
	80	89.83	5.27	97.53	7.77	94.80	5.80
Neochlorogenic acid	3.2	93.15	8.94	87.53	12.96	99.44	8.05
	16	93.43	3.48	85.10	4.01	94.46	4.31
	80	88.19	4.08	87.22	2.81	107.42	7.15
Cryptochlorogenic acid	3.2	105.91	7.83	105.20	7.04	103.20	7.52
	16	102.46	4.00	99.17	3.64	98.88	3.03
	80	104.57	2.86	85.19	9.96	92.08	6.50
Liquiritin apioside	0.64	107.40	9.62	97.06	11.49	110.25	5.70
	16	109.36	2.66	114.91	2.33	104.39	0.87
	80	108.55	1.41	102.01	6.41	87.64	7.98
Luteoloside	0.64	110.17	3.38	114.49	4.56	102.64	2.70
	16	113.76	3.76	110.10	2.86	100.41	0.63
	80	110.37	3.60	98.89	4.84	85.33	2.71
Isochlorogenic acid C	3.2	94.25	13.83	87.19	11.87	87.48	8.84
	16	97.06	3.91	96.46	10.10	92.55	8.19
	80	91.80	4.24	92.85	3.81	87.10	3.87
Salvianolic acid B	16	104.60	2.04	104.22	2.46	107.95	4.87
	80	87.42	5.79	86.85	2.89	93.77	4.54
	400	88.54	3.97	88.49	6.10	103.84	9.20
Luteolin	0.32	98.58	2.91	101.07	5.13	96.27	6.37

**Analyte**	**Spiked (ng/mL)**	**Short-term**		**Long-term**		**Three-freeze-thraw**	
		**Accuracy（%）**	**RSD（%）**	**Accuracy（%）**	**RSD（%）**	**Accuracy（%）**	**RSD（%）**
	1.6	109.94	3.62	108.14	2.34	105.68	3.20
	16	111.22	1.78	102.76	4.14	87.80	2.39
	0.064	87.36	4.12	98.84	7.42	91.09	5.24
Isoliquiritigenin	1.6	94.23	2.14	89.37	3.52	107.05	6.42
	16	101.97	4.08	104.72	2.54	92.87	4.56
	0.64	85.71	7.16	86.55	7.69	87.23	8.17
Formononetin	16	95.00	1.69	93.09	2.42	98.05	1.91
	80	94.21	1.19	97.80	3.22	87.47	2.02
	16	105.83	11.28	96.56	9.95	94.09	13.28
Astragaloside IV	40	111.74	3.23	114.57	1.88	114.83	3.79
	80	105.43	4.38	113.57	7.55	112.76	6.05

Table S11 Matrix effect and recovery data for three analytes (*n* = 6)

Analyte	Spiked (ng/mL)	Matrix effect		Recovery	
		Accuracy （%）	RSD （%）	Accuracy （%）	RSD （%）
	0.64	98.68	14.08	90.72	8.20
Chlorogenic acid	16	89.53	3.81	92.87	3.63
	80	91.33	4.31	85.81	9.07
	3.2	100.72	14.82	108.83	10.66
Neochlorogenic acid	16	102.33	3.74	94.51	5.21
	80	106.65	3.39	87.40	9.26
	3.2	107.50	8.42	91.97	3.70
Cryptochlorogenic acid	16	105.26	3.44	90.70	4.31
	80	105.82	3.92	93.42	11.42
	0.64	87.39	3.23	106.17	4.66
Liquiritin apioside	16	85.99	2.51	101.89	5.00
	80	85.21	0.98	102.36	7.34
	0.64	87.16	12.70	88.98	8.65
Luteoloside	16	85.12	2.52	103.55	3.93
	80	88.30	0.76	110.42	14.42
	3.2	87.46	8.17	93.00	6.48
Isochlorogenic acid C	16	92.62	4.30	98.26	9.46
	80	94.99	7.23	89.42	11.30
	16	105.84	13.04	85.99	2.42
Salvianolic acid B	80	101.26	6.57	85.98	3.72
	400	100.27	13.42	88.61	12.90

**Analyte**	**Spiked (ng/mL)**	**Matrix effect**		**Recovery**	
		**Accuracy （%）**	**RSD （%）**	**Accuracy （%）**	**RSD （%）**
	0.32	86.56	4.43	102.09	3.02
Luteolin	1.6	92.65	2.78	92.41	3.19
	16	90.27	3.57	103.54	12.52
	0.064	89.24	4.34	114.00	5.01
Isoliquiritigenin	1.6	100.20	5.01	95.23	3.32
	16	108.04	5.22	95.32	5.24
	0.64	98.59	3.69	114.73	3.53
Formononetin	16	87.23	2.62	105.24	3.02
	80	87.45	5.05	109.30	9.33
	16	108.33	9.55	101.35	6.88
Astragaloside IV	40	95.88	13.47	104.36	3.11
	80	102.06	1.76	109.31	8.62

Table S12 Oral relative bioavailabilities of components in QSG

Compound	$AUC_{0\rightarrow\infty}$ （ng h/mL）	Dosage (mg)	Oral relative bioavailability (%)
Chlorogenic acid	175.7	20.544	0.006
Neochlorogenic acid	1418	24.849	0.040
Cryptochlorogenic acid	576.2	15.618	0.026
Liquiritin apioside	185.7	13.903	0.009
Luteoloside	20.86	0.789	0.019
Isochlorogenic acid C	170.5	15.093	0.008
Salvianolic acid B	2176	117.023	0.013
Isoliquiritigenin	4.891	0.063	0.055
Formononetin	27.66	0.591	0.033
Astragaloside IV	144.9	0.950	0.107
